# In Vivo *versus* Augmented Reality Exposure in the Treatment of Small Animal Phobia: A Randomized Controlled Trial

**DOI:** 10.1371/journal.pone.0148237

**Published:** 2016-02-17

**Authors:** Cristina Botella, M. Ángeles Pérez-Ara, Juana Bretón-López, Soledad Quero, Azucena García-Palacios, Rosa María Baños

**Affiliations:** 1 Department of Basic Psychology, Clinic and Psychobiology, Universitat Jaume I, Castellón, Spain; 2 Department of Personality, Evaluation and Psychological Treatment, Universitat de València, Valencia, Spain; 3 CIBER Fisiopatología Obesidad y Nutrición (CIBERObn), Instituto Salud Carlos III, Madrid, Spain; Erasmus University Rotterdam, NETHERLANDS

## Abstract

Although in vivo exposure is the treatment of choice for specific phobias, some acceptability problems have been associated with it. Virtual Reality exposure has been shown to be as effective as in vivo exposure, and it is widely accepted for the treatment of specific phobias, but only preliminary data are available in the literature about the efficacy of Augmented Reality. The purpose of the present study was to examine the efficacy and acceptance of two treatment conditions for specific phobias in which the exposure component was applied in different ways: In vivo exposure (N = 31) versus an Augmented Reality system (N = 32) in a randomized controlled trial. “One-session treatment” guidelines were followed. Participants in the Augmented Reality condition significantly improved on all the outcome measures at post-treatment and follow-ups. When the two treatment conditions were compared, some differences were found at post-treatment, favoring the participants who received in vivo exposure. However, these differences disappeared at the 3- and 6-month follow-ups. Regarding participants’ expectations and satisfaction with the treatment, very positive ratings were reported in both conditions. In addition, participants from in vivo exposure condition considered the treatment more useful for their problem whereas participants from Augmented Reality exposure considered the treatment less aversive. Results obtained in this study indicate that Augmented Reality exposure is an effective treatment for specific phobias and well accepted by the participants.

## Introduction

Specific phobia (SP) is the most prevalent 12-month disorder DSM–IV disorder (8.7%) [1] among the USA population, whereas lower prevalence were found in some European countries (3.5%) [2]. In the case of the animal phobia subtype [3], the prevalence ranges between 3.3% and 7% [3,4] and it is one of the most prevalent subtypes of SP [5,6]. Around 50–80% of people with a SP have one or more comorbid mental disorders [4,6,7], or even multiple phobias [8], which cause serious interference in daily life [4].

Currently, the treatment of choice for SP (including small animal phobia) is in vivo exposure [9,10]. However, there are several difficulties in the application of this technique. In fact, only 7.8% of people suffering from phobias seek treatment [11], and only 0.8% receive specific treatment for this disorder [12]. This may be due to the lack of evidence-based treatments offered by the health care system, the long waiting lists, or the fact that many therapists are not well trained in applying exposure therapy [13–15]. Second, although there is empirical evidence of the efficacy of exposure therapy, this treatment has a “public relations” problem [16]. Some studies have found that therapists may be reluctant to provide exposure-based therapy [17,18] because they consider it cruel and at odds with some ethical considerations [16,19]. In fact, [20] they reported that only 17% of a sample of 852 practitioners used exposure in the treatment of PTSD. Although the empirical data do not support such ideas and it has been demonstrated that exposure therapy is effective and safe and it does not produce higher dropout rates than other forms of therapy [21], still some patients and therapists may be reluctant to receive this technique [22,23]. García-Palacios, Botella, Hoffman, and Fabregat [24], explored the preferences of a sample of people suffering specific phobias when offering the usual way of delivering exposure (in vivo) versus exposure delivered with virtual reality (VR) and 76% of patients chose VR exposure. Furthermore, in some cases other limitations are present, such as lack of confidentiality or treatment-associated costs when it is necessary to work outside the therapist’s office [15,25]. Thus, new models for delivering therapy could be useful to improve the opinions about exposure therapy and to bring evidence-based techniques to those who need them and reduce the general impact of the burden of mental illness worldwide [13].

In recent years, the use of Information and Communication Technologies (ICT’s) for the treatment of psychological disorders has increased considerably. One way of improving the effectiveness or clinical utility of psychological treatment using ICT devices comes from Virtual Reality (VR). Specifically, VR has been shown to be an effective tool for applying the exposure component in the treatment of anxiety [26–30]. Specifically, the meta-analysis conducted by Power and Emmelkamp [29] demonstrated that In vivo exposure (IVE) is not significantly more effective than VR exposure (VRE) for various anxiety disorders (including SP). In the case of SP, the data on the treatment of spider phobia show that VRE is effective for this problem in adults [31] and children [32], more effective in comparisons with wait-list control groups [33,34] and as effective as in vivo exposure [35]. VR is also well accepted [24,36,37], even in children [38].

Augmented Reality (AR) is a variant of VR that combines the real world with virtual elements, using computer graphics mixed with the real world in real time [39]. In AR, the person sees an image composed of a visualization of the real world and a series of virtual elements, which, at the same time, are super-imposed on the real world. The most important aspect of AR is that the virtual elements supply the person with relevant information that is not found in the real world.

The literature shows great advances in AR applications in other fields [40–42]. In the psychological treatment field, AR presents the same advantages as VR (that is, total control over the way the exposure is conducted, easier access to the threatening stimuli, no risk of real danger to the patient, the possibility of going beyond reality, confidentiality), but it can be less expensive than VR because it is not necessary to model the whole environment.

The first study using AR for the treatment of SP was carried out by Botella and colleagues [43]. It consisted of a case study of a woman who suffered from cockroach phobia. The exposure was applied using AR and following the guidelines of Intensive One-Session-Treatment (OST) [44,45], a well-established procedure for the treatment of SP [46–48]. Results from this case study [43] showed a decrease in scores on fear and avoidance and on the variables related to the BAT. Specifically, after the AR exposure session, the participant was capable of interacting with real cockroaches, and the improvements were maintained at the 1-month follow-up. Regarding the participant’s opinion of the AR exposure treatment, she reported a high level of satisfaction, considering AR to be slightly aversive, but less aversive than in vivo exposure. This same AR system has been shown to be useful for inducing anxiety in participants [49], and it has also been tested using a multiple baseline design with 6 individuals who suffered from cockroach phobia [50]. Results showed that AR exposure was effective, as all the participants improved significantly on all the outcome measures. Furthermore, the improvements were maintained over time (6- and 12-month follow-ups).

These preliminary studies show that exposure through AR can be useful for the treatment of SP (cockroaches and spiders). However, despite these promising results, we have found no randomized controlled trials on AR efficacy compared to the current treatment of choice for SP, in vivo exposure, nor any study focused on analyzing the opinion and acceptance by the participants of AR exposure.

Thus, the present study aims to examine the efficacy and acceptance (expectations and satisfaction) of two treatment conditions in which the exposure component was applied in different ways: in vivo (IVE) versus the AR system (ARE), in a randomized controlled trial. Taking into account the meta-analysis data in the literature on IVE, we expect this treatment to be efficacious. Data from previous AR pilot tests lead us to predict that this procedure will also be effective. As for the participants’ acceptance, we expect both treatments to be well accepted, with AR being considered less aversive than IVE.

## Materials and Methods

### Design

The study was registered in the National Institute of Health Registration System (http://www.clinicaltrials.gov) with Clinical Trials Registration Number: NCT01361074. The authors confirm that all ongoing and related trials for this intervention are registered. The RCT was conducted in accordance with the CONSORT 2010 Statement [51] and the CONSORT-EHEALTH guidelines [52,53] to study the efficacy of two experimental conditions: 1) In Vivo Exposure and 2) Augmented Reality Exposure (see S1 CONSORT Checklist). The participants were randomly assigned to the two experimental conditions. Repeat measurements at pre-treatment, post-treatment, three-month follow-up, and six-month follow-up were included. Regarding the sample size, power calculations were based on data from two meta-analyses [28,29]. Both of them were based on studying the efficacy of VR for the treatment of anxiety disorders, and both included the BAT as outcome measure. Calculations indicated that a sample size of 15 participants in each group (30 in total) would be sufficient to detect an effect size of d = 1.11 or d = 1.12 with a power of 80%; however, we were more conservative and decided to double this estimation and include at least 30 participants in each treatment condition (at least 60 in all). This decision was made for two main reasons: first, although data were available from meta-analyses on VR efficacy for the treatment of SP using several VR sessions, there were no data available from RCT about the efficacy of VR or AR applied in a single session that could serve as a reference. Therefore, we decided to be more conservative and increase the sample size in order to have more power and try to avoid a Type II error.

### Participants, recruitment and randomization

The study was approved by the Ethical Committee of the Jaume I University (*Comisión Deontológica de la Universitat Jaume I*) at January 2011 (see S1–S3 Files). The recruitment processes as well as the data collection took place at January 2011 to January 2013. Participants were recruited through advertisements sent by mail to university community members and announcements placed around the campus and in the local media.

Inclusion criteria were: (a) meeting DSM-IV-TR [5] for the diagnosis of SP (animal subtype) to cockroaches or spiders; (b) being at least 18 years old and having a minimum 1-year duration of the phobia; (c) being willing to follow the study conditions and sign the consent form; and d) presenting a score of at least 4 on the fear and avoidance scales of the diagnostic interview applied. Exclusion criteria were: (a) having another psychological problem that requires immediate attention; (b) having current alcohol or drug dependence or abuse, psychosis or severe organic illness; (c) currently being treated in a similar treatment program; (d) being capable of inserting their hands in a plastic container with a cockroach or a spider (during the behavioral test); and (e) taking anxiolytics during the study (or in the case of taking them, changing the drug or dose during the study).

A total of 63 participants who meet the eligibility criteria were included in the study.

The randomization of the participants took place after assessing the eligibility criteria. The person responsible for the randomization was an independent researcher with no clinical involvement in the trial and no access to the study data. She assigned participants to either the IVE (N = 31) or ARE (N = 32) condition, based on a computer-generated randomization list created by the “Random Allocation Software”; version 1.0. Therapists and participants involved in the trial were blind to treatment allocation during the assessment.

### Measures

The assessment protocol included diagnostic, main outcome, and secondary measures to assess the main features of the spider and cockroach phobias, interference and severity measures as well as expectations and satisfaction regarding the exposure treatment. In this paper, the most relevant measures are presented.

#### Primary outcome measures

Behavioral Avoidance Test (BAT; Adapted from Öst, Salkovskis, and Hellström’s [54]). The BAT is an observational measure used to assess the features of the phobia in a context of exposure to the feared object, in order to obtain objective data about the person’s fear. For this study, a container containing a live cockroach or spider was placed 5 meters from the entrance to a room. Then, participants were asked to enter the room and come as close to the animal as possible. Before the test, the therapist asked them about their level of anxiety, avoidance and belief related to the fear on a scale from 0 to 10, where 0 is “no fear”, “no avoidance” and “does not believe the content of the thought at all”), and 10 is “extreme fear”, “total avoidance” and “believe the thought is totally true”. Their performances on the test were scored, transforming the distance into a score rated on a scale from 0 to 12, where 0 = “refuses to enter the room” and 12 = “The participant interacts by holding the animal on a post card for more than 20 seconds”. The maximum anxiety experienced by the participant during the BAT performance and the severity of the fear assessed by the therapist are also measured on a scale from 0 to 10, where 0 = “no fear” and 10 = “severe fear”. This measure was used in a previous study where a more detailed description was provided [50].

#### Secondary Outcome Measures

Fear of Spiders Questionnaire (FSQ; adapted from Szymanski and O’Donohue [55]). This is a self-report questionnaire containing 18 items about spiders and designed to assess the severity of the phobia. Each item is answered on a Likert scale ranging from 0 (“I strongly disagree”) to 7 (I strongly agree). Scores can range from 0 to 126. Muris and Merckelbach [56] found that the mean score in a group of people before treatment was 89.1 (SD = 19.6) and after treatment 39.9 (SD = 25.4). In the same study, the mean score of control subjects without spider phobia was 3.0 (SD = 7.8). The FSQ has excellent psychometric properties. To evaluate cockroach phobia, we used an adaptation in the Spanish population [57].

Spider Phobia Beliefs Questionnaire (SBQ; adapted from Arntz, Lavy, van der Berg and van Rijssoort [58]). This is a self-report questionnaire composed of 78 items divided into two scales: fear beliefs related to spiders (SBQ-1) and the person’s reaction to their presence (SBQ-2). All items are scored on a scale from 0 to 100, where 0 means "I do not believe it at all" and 100 means "I totally believe it." Regarding the psychometric properties reported by [58], on the one hand, the average scores on subscale SBQ-1 in clinical samples were 48.76 before treatment (SD = 17.74) and 10.15 (13.69) after treatment; on the other hand, the mean scores on subscale SBQ-2 were 49.79 (SD = 18.72) before treatment and 8.00 (SD = 13.15) after treatment. As for reliability, good results are reported for internal consistency and test-retest reliability. The SBQ was also adapted to evaluate cockroach phobia in the Spanish population [59].

Fear and Avoidance Scales (Adapted from Marks and Mathews [60]). Along with their therapists, the participants established the situations related to cockroaches and spiders that caused them the most fear and distress (e.g. taking out the trash at night), as well as their negative thoughts associated with them (e.g. If I see cockroaches I’ll go crazy; The cockroaches are going to jump on me). They then used scales from 0 to 10 to assess their degree of fear (0 = “no fear”; 10 = “extreme fear”) and avoidance (0 = “never avoid”; 10 = “always avoid”) for each feared situation. In addition, the degree of belief in the negative thoughts related to the target behaviors was also assessed on a scale ranging from 0 (“I do not believe the content of the thought at all”) to 10 (“I believe the thought is totally true”). For this study, the most significant target behavior chosen by each participant was used (Main Target Behaviour, MTB).

Diagnostic Status. The Anxiety Diagnostic Interview Schedule IV (ADIS-IV-L [61]) is a semi-structured interview used to determine the diagnostic status and quantify the different features related to the phobia, such as the fear and avoidance rates, on a scale from 0 to 8 (0 = no fear, no avoidance; 8 = extreme fear, extreme avoidance), and the interference and distress perceived by the participant (on a scale from 0 to 8, where 0 = “not at all” and 8 = “Very severe). The ADIS-IV has shown adequate psychometric properties according to Antony, Orsillo, and Roemer [62], with an inter-rater reliability ranging from satisfactory to excellent when used by expert clinicians familiar with the DSM diagnostic criteria [63].

Clinician Severity Scale (CSS; [64]). At the end of the individual interviews, the clinician rated the severity of the patient’s phobias on a scale from 0 to 8, where 0 = “symptom free” and 8 = “extremely severe and disabling, all aspects of life are affected”. This scale was used in previous studies to assess spider phobia [33].

Expectations and satisfaction regarding the exposure treatment (adapted from Borkovec and Nau, [65]). This questionnaire measures the participants’ expectations for the exposure component before treatment and their satisfaction with it after treatment. It includes six items rated from 0 (‘not at all’) to 10 (‘very much’) to address how logical, satisfactory, recommendable, useful for other problems and for the patient’s problem, and how aversive the treatment is. The adaptation of these scales has been used in previous studies [66,67].

### Augmented Reality System and Hardware

**Fig 1:**
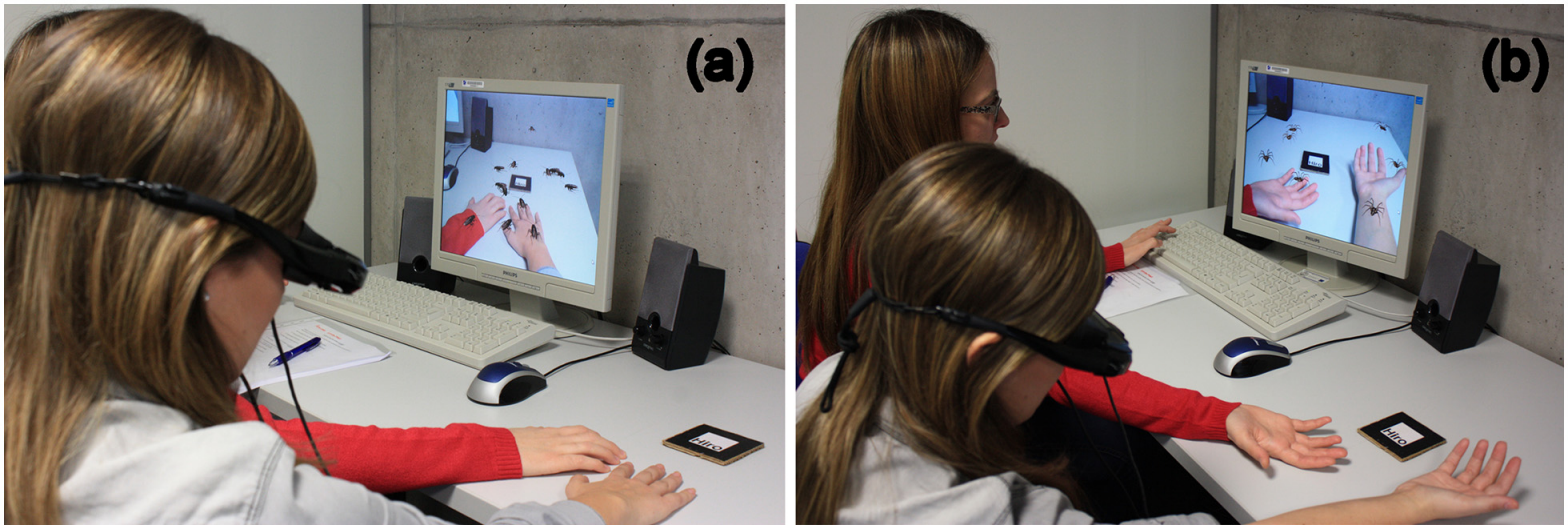
Use of the AR system during the exposure session (simulation). (a) virtual cockroaches; (b) virtual spiders.

In the present work, two devices were used to display Mixed Reality images: 1) AR 5DT HMD (head-mounted display) with a 800 x600 resolution and a high (40 degrees) fields of view where an USB Creative NX-Ultra camera is attached to the HMD to capture video stream; and 2) *VR Goggles* (Vuzix) that include two LCD devices with a 640x480 resolution and a 30-degree field of view and an embedded camera. The system includes 3D spiders and cockroaches and enables real-time interactivity, so that participants can see the actual and real place where they are through the display device, and the feared stimuli (spiders and cockroaches) in the same place (Fig 1).

**Fig 2:**
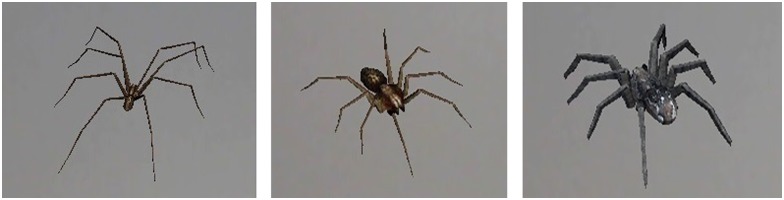
Types of spiders available into the AR system.

Bodies and movements of the spiders and cockroaches were modelled using 3DStudio and exported in VRML format; their movements and texture were similar to those of real spiders and cockroaches. Modulating variables that can be manipulated in the AR System are the following: number of animals, movements of the animals, their size (small, medium and large), type of spider (Fig 2), and, finally, the possibility of displaying the animal on various surfaces (e.g., on the table, on the floor, on the person, etc.).

All of these combined options enable the therapist to apply the treatment progressively.

**Fig 3:**
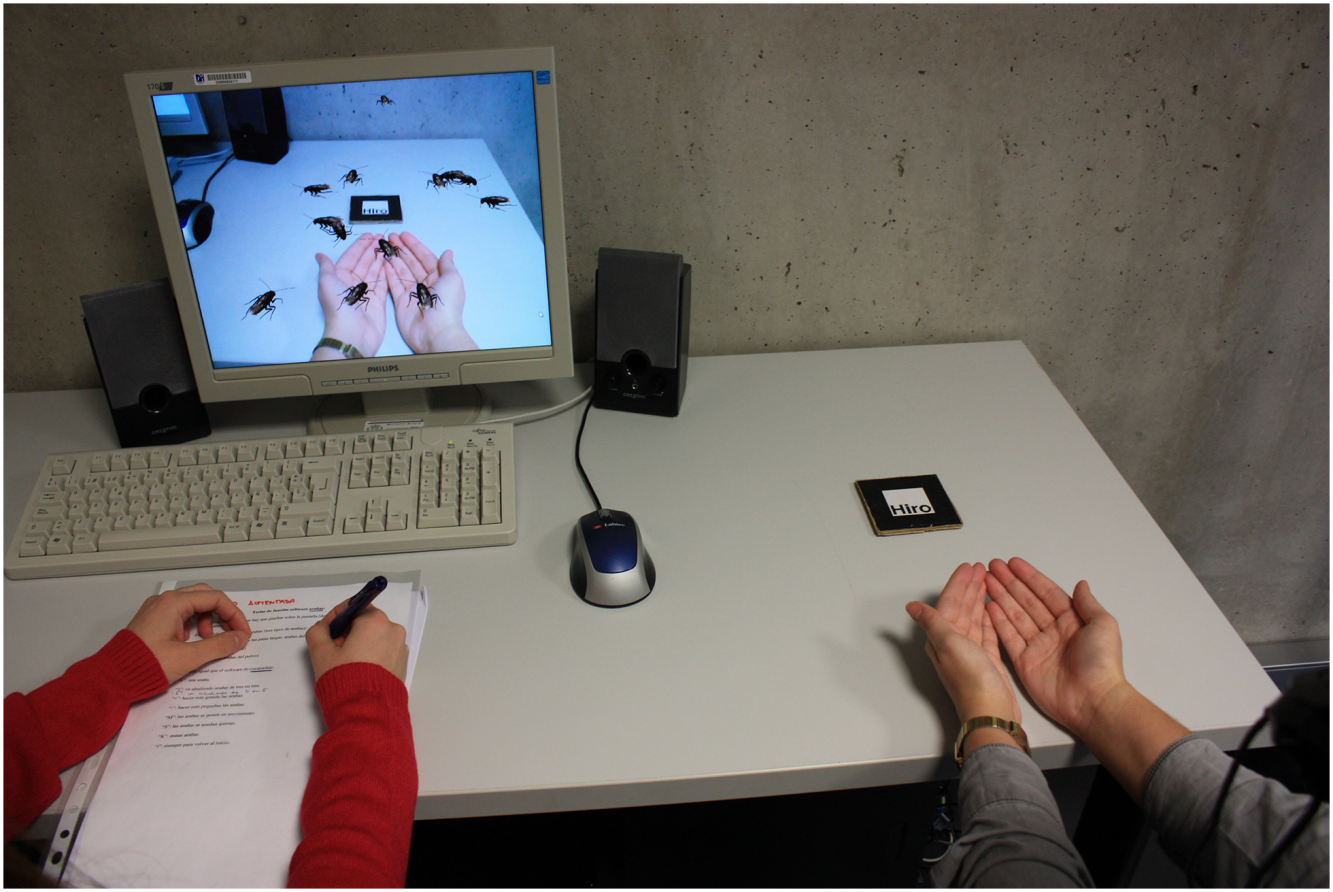
A participant interacting with the virtual cockroaches in her hands.

In Fig 3, we can see a person interacting with the cockroaches with her hands. A full description of the system can be found in previous studies [43,50].

### Treatment

We used exposure therapy according to the “one-session treatment” guidelines developed by Öst et al. [44]. The treatment program included several therapeutic components applied in only one individual session lasting up to 3 hours. The components, based on Öst [45], were exposure to the feared object (cockroaches or spiders), modelling, reinforced practice, and cognitive challenge. The focus of the treatment was for participants to face phobic situations in a controlled, graduated and planned way in order to tolerate the fear experienced, which would allow them to disconfirm the negative thoughts related to the presence of the feared object and its consequences, and prevent cognitive and behavioral avoidance in a safe context. The therapist’s instructions to the participants followed the recommendations of Öst, Salkovskis, and Hellström [54]: his/her role is to encourage the participant and collaborate with him/her to advance in the treatment objectives. Moreover, once the treatment was over, participants were advised to continue their exposure to the feared animals in order to generalize the results to other situations, although they were not given guided instructions for this exposure.

**Fig 4:**
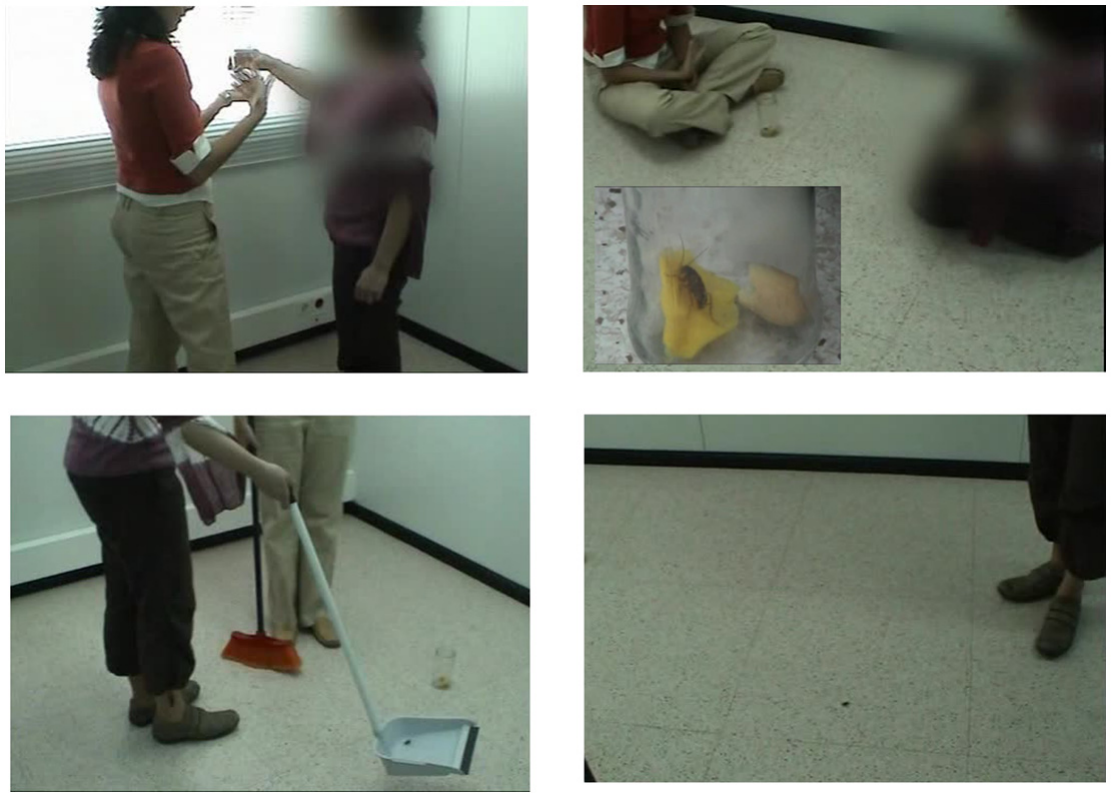
A participant exposed to real cockroaches.

This treatment was applied in two different ways:

- IVE condition: Participants in the IVE condition were exposed to real small animals, that is, live cockroaches or spiders (see Fig 4)
- ARE condition: Participants were exposed to virtual animals (cockroaches or spiders) using the AR system (see Figs 1 and 3).

### Therapists

Five therapists were involved in the study, all of whom had a PhD or a Master’s degree in Psychology. All of them were trained in CBT and had extensive experience in the treatment of anxiety disorders and in the exposure technique (either in vivo or VR/AR). In addition, they received training in this treatment protocol for senior clinicians. Depending on the availability of therapists, in certain cases the clinician who performed the initial assessment was different from the one who conducted the treatment, while in other cases the clinician was the same; the same thing occurred in follow-up assessments. In addition, it is important to point out that all the therapists conducted both the IVE and ARE treatments.

All the therapists were supervised by senior clinicians with PhDs in weekly sessions. Moreover, all of the assessment and treatment sessions were video recorded in order to supervise each therapist’s performance.

### Procedure

The study took place at the Emotional Disorders Clinic at Jaume I University. The assessment protocol was conducted in one session lasting one-and-a-half hours. During this session, after the eligibility criteria had been confirmed, participants were informed and asked to participate in the study. If they agreed to participate, they signed the consent form. Then, they were randomly assigned to one of the two experimental treatment conditions (IVE or ARE) and continued to complete the rest of the assessment. The treatment was carried out in one intensive IVE or ARE session (up to three hours). Finally, after completing the treatment session, all the participants were again assessed at post-treatment (on the same day) and at 3- and 6-month follow-ups.

To control procedural fidelity, the assessment and treatment protocol were available. In fact, specific instructions for conducting both conditions (IVE or ARE) were presented, based on the OST protocol [44,45,54]. In addition, as mentioned above, all the therapists were supervised by senior clinicians and video recorded in order to control compliance with the procedure.

### Statistics and data analysis

The Chi-square test was conducted to evaluate group differences in demographic variables (gender, sex, level of studies and marital status). In addition, Student’s “t” test was used to test differences between the two treatment conditions at pre-treatment on all of the efficacy measures and treatment acceptance, as well as other variables, such as participants’ age and duration of the phobia. All post-treatment and follow-up analyses involve a conservative intention-to-treat (ITT; [68]) design, where missing data were addressed by carrying forward the last available data (last observation carried forward model; LOCF). Furthermore, data considering the completer participants were also analyzed.

In order to test the treatment efficacy in both conditions repeated-measures ANOVAs were used to compare the time effect on the measures at four assessment times (pre, post, 3- and 6-month follow-ups) and the time interaction among the treatment conditions at pre, post, 3- and 6-month follow-ups. Effect sizes (Cohen’s d) were calculated for within- and between-group changes, from pre-treatment to post-treatment, from pre-treatment to 3-month follow-up, and from pre-treatment to 6-month follow-up. Cohen’s d [69] is based on the pooled standard deviation, and effect sizes were categorized as no effect (< 0.2), small effect (0.2–0.5), medium effect (0.5–0.80) or large effect (> 0.80). Finally, Chi-squared tests were performed in order to examine the differential clinically significant improvement rates, based on the Jacobson and Truax indexes [70] for FSQ and SBQ scores at post-treatment and 3- and 6-month follow-ups in the two treatment conditions. In this study, the classification proposed by Iraurgi [71] and Kupfer [72] was used, where “Recovered” means the participant is situated in the normal or functional distribution; “Improved” indicates a significant improvement, but not situated in the normal or functional distribution; “No change” means no significant improvement and not situated in the normal or functioning distribution; and finally, “Deteriorated” indicates a significant deterioration.

All statistical analyses were performed using SPSS 20.0.

## Results

### Participant flow and attrition

**Fig 5:**
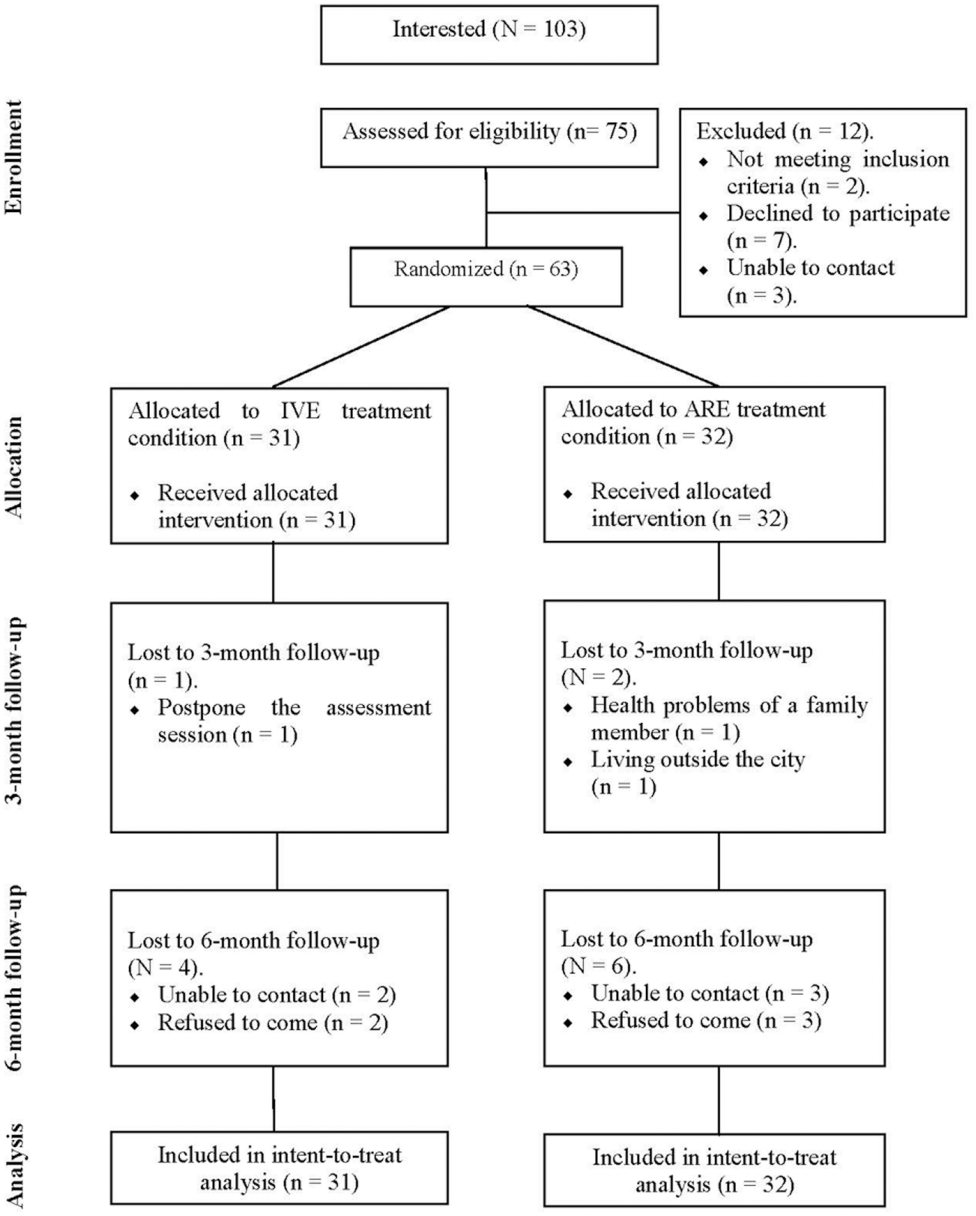
Flow diagram of study participants, point of random assignment, and dropouts in each stage of the study.

A complete description of the participants’ attrition is shown in Fig 5.

Initially, 103 people contacted the Emotional Disorders Clinic at Jaume I University to show their interest in taking part in the study. Out of these 103 people, 75 were assessed for eligibility criteria. Twelve participants were excluded from the study before treatment. The reasons for their exclusion were: they did not meet the inclusion criteria (N = 2), they refused to attend (N = 7), or we were unable to contact them again (N = 3). Finally, based on the DSM-IV-TR criteria [7], 63 participants were included in the study.

As the flowchart reveals, none of the participants withdrew from either treatment condition after randomization or during the treatment session. In addition, all of them completed the post-treatment assessment. At the 3-month follow-up, a total of 60 participants attended the assessment session, 30 participants in the IVE condition (96.77%) and 30 in the ARE condition (93.75%). At the 6-month follow-up, a total of 53 participants attended the assessment session, 27 participants in the IVE condition (87.10%) and 26 participants in the ARE condition (81.25%). No significant differences in attrition rates were found between the treatment conditions.

### Baseline data and pre-treatment differences

#### Demographic and clinical characteristics

Descriptive data collected about demographic variables, diagnosis, phobia’s duration, and medication are shown in Table 1.

**Table 1:** Descriptive data about demographic variables, diagnosis, phobia’s duration, and medication.

	IVE	ARE	Total
**Age**			
Range	21–70	20–57	20–70
Mean (SD)	32.45 (11.50)	31.03 (10.08)	31.73 (10.74)
**Sex**			
Men	1 (3.2%)	3 (9.4%)	4 (6.3%)
Woman	30 (96.8%)	29 (90.6%)	59 (93.7%)
**Marital status**			
Single	14 (45.2%)	17 (53.1%)	31 (49.2%)
Married/ partnered	15 (48.4%)	14 (43.8%)	29 (46%)
Divorced	2 (6.5%)	1 (3.1%)	3 (4.8%)
**Level of studies**			
Elementary school	3 (9.7%)	0 (0%)	3 (4.8%)
High school	3 (9.7%)	4 (12.5%)	7 (11.1%)
University degree	25 (80.6%)	28 (87.5%)	53 (84.1%)
**Diagnosis**			
Cockroaches phobia	27 (87.1%)	27 (84.4%)	54 (85.7%)
Spiders phobia	4 (12.9%)	5 (15.6%)	9 (14.3%)
**Phobia’s duration (years)**			
**Mean (SD)**	18.78 (12.87)	17.68 (13.94)	18.21 (13.33)
**Medication**			
Anxiolytics	2 (3.17%)	0 (0.00%)	2 (3.17%)
Antidepressants	0 (0.00%)	0 (0.00%)	0 (0.00%)

As the table shows, most of the sample were women (93.7%) and had a diagnosis of cockroach phobia (85.7%). The mean age was 31.73 (SD = 10.74), ranging from 20 to 70 years. The sample was practically divided between married and single, and most of them had a university degree (84.1%). The average length of the phobia was 18.21 years, and only 2 participants were taking anxiolytic drugs. One participant took medication only occasionally due to a previous diagnosis of agoraphobia, but he/she did not take it during the treatment. The other person remained at the same dose throughout the study without any changes in the type of drug. In the case of this participant, she was taking Alprazolam in the framework of a previous eating disorder (in order to control binge-eating episodes).

#### Pre-treatment differences

Statistical analyses showed no differences between the two groups at pre-treatment on any demographic variables, phobia duration, medication or diagnostic variables (cockroach or spider phobia).

Regarding primary and secondary clinical variables, no differences were found between the two experimental conditions on any of these measures at pre-treatment. Means and SDs are shown in Table 2.

**Table 2:** Means, standard deviations, within-group and between-group effect sizes for the ITT analysis of all outcome measures at pre-post and 6 month follow-up.

Measure		Group	Pre-treatment	Post-treatment	6-month FU	Within-group effect size pre-post treatment	Within-group effect size pre-6-month FU	Between group effect size at post treatment	Between group effect size at 6-month FU
			Mean (SD)	Mean (SD)	Mean (SD)	*d* (95% CI)	*d* (95% CI)	*d* (95% CI)	*d* (95% CI)
**BAT**	Fear-BAT								
		IVE	8.39 (2.32)	3.16 (3.08)	3.65 (3.19)	1.95 (1.28 to 2.62)	1.73 (1.04 to 2.41)	-0.93 (-1.62 to -0.24)	-0.18 (-0.10 to 0.63)
		ARE	8.62 (2.12)	5.75 (2.59)	4.25 (3.52)	1.23 (0.66 to 1.80)	1.53 (0.83 to 2.23)		
	Avoidance-BAT								
		IVE	8.87 (2.14)	2.94 (3.49)	3.52 (4.03)	2.08 (1.37 to 2.79)	1.69 (0.89 to 2.48)	-0.80 (-1.61 to 0.00)	-0.11 (-1.13 to 0.92)
		ARE	8.50 (2.05)	5.56 (3.15)	3.97 (4.39)	1.12 (0.48 to 1.76)	1.34 (0.52 to 2.17)		
	Belief-BAT							
		IVE	8.42 (2.92)	3.03 (3.07)	3.00 (3.62)	1.83 (1.09 to 2.56)	1.67 (0.87 to 2.48)	-0.28 (-1.00 to 0.45)	0.04 (-0.78 to 0.85)
		ARE	8.63 (2.22)	3.84 (2.89)	2.88 (3.08)	1.89 (1.27 to 2.51)	2.18 (1.53 to 2.82)		
	Performance-BAT								
		IVE	4.10 (2.20)	10.13 (1.94)	10.16 (2.28)	-2.95 (-3.46 to -2.45)	-2.75 (-3.29 to -2.20)	0.63 (0.05 to 1.22)	0.36 (-0.35 to 1.07)
		ARE	4.09 (2.04)	8.63 (2.79)	9.13 (3.44)	-1.89 (-2.48 to -1.30)	-1.81 (-2.49 to -1.13)		
	Maximum anxiety-BAT								
		IVE	7.10 (1.92)	3.42 (2.55)	3.26 (2.57)	1.66 (1.10 to 2.21)	1.72 (1.16 to 2.28)	-0.43 (-1.06 to 0.20)	-0.28 (-0.96 to 0.39)
		ARE	7.06 (1.76)	4.53 (2.64)	4.03 (2.95)	1.15 (0.60 to 1.69)	1.27 (0.68 to 1.85)		
	Severity-BAT (Clinician)								
		IVE	5.77 (1.22)	2.40 (1.63)	2.26 (1.86)	2.38 (2.03 to 2.73)	2.27 (1.88 to 2.65)	-0.44 (-0.88 to 0.01)	-0.25 (-0.79 to 0.30)
		ARE	5.72 (1.20)	3.19 (2.01)	2.81 (2.57)	1.55 (1.15 to 1.95)	1.47 (0.99 to 1.96)		
**FSQ**		IVE	95.81 (13.96)	39.71 (23.10)	38.13 (29.19)	2.99 (-1.68 to 7.66)	2.56 (-3.04 to 8.16)	-0.22 (-5.62 to 5.19)	-0.08 (-7.73 to 7.76)
		ARE	94.09 (17.99)	44.47 (21.38)	40.75 (33.54)	2.55 (-2.21 to 7.32)	2.01 (-4.48 to 8.50)		
**SBQ**	SBQ-1								
		IVE	52.49 (13.70)	18.20 (11.87)	14.95 (15.57)	2.72 (-0.42 to 5.86)	2.60 (-0.99 to 6.19)	-0.27 (-3.66 to 3.12)	0.02 (-4.17 to 4.22)
		ARE	49.42 (18.00)	21.93 (15.73)	14.56 (18.78)	1.65 (-2.42 to 5.73)	1.92 (-2.51 to 6.36)		
	SBQ-2								
		IVE	39.92 (18.63)	9.23 (8.18)	11.14 (14.98)	2.17 (-1.35 to 5.69)	1.73 (-2.41 to 5.87)	-0.23 (-2.82 to 2.35)	-0.10 (-4.47 to 4.27)
		ARE	38.97 (20.92)	11.65 (12.55)	12.91 (20.50)	1.61 (-2.55 to 5.77)	1.28 (-3.72 to 6.27)		
**MTB**	Fear-MTB								
		IVE	8.65 (1.47)	4.10 (2.40)	2.58 (2.88)	2.32 (1.84 to 2.81)	2.70 (2.14 to 3.26)	-0.20 (-0.81 to 0.42)	-0.44 (-1.17 to 0.28)
		ARE	8.78 (1.31)	4.59 (2.65)	3.88 (3.07)	2.04 (1.53 to 2.54)	2.11 (1.54 to 2.68)		
	Avoidance-MTB								
		IVE	9.10 (1.70)	2.42 (2.29)	1.97 (3.21)	3.37 (2.87 to 3.86)	2.82 (2.19 to 3.45)	-0.65 (-1.32 to 0.03)	-0.34 (-1.18 to 0.49)
		ARE	8.78 (1.62)	4.19 (3.19)	3.13 (3.62)	1.84 (1.23 to 2.45)	2.05 (1.37 to 2.72)		
	Belief -MTB								
		IVE	8.58 (2.13)	3.26 (2.22)	2.71 (2.92)	2.49 (1.95 to 3.02)	2.33 (1.71 to 2.96)	-0.24 (-0.87 to 0.39)	-0.20 (-0.91 to 0.51)
		ARE	8.78 (1.31)	3.88 (2.93)	3.28 (2.92)	2.19 (1.65 to 2.74)	2.47 (1.92 to 3.01)		
**CSS**		IVE	5.62 (1.08)	2.69 (1.44)	2.31 (1.81)	2.34 (2.03 to 2.65)	2.26 (1.89 to 2.62)	-0.30 (-0.66 to 0.05)	-0.26 (-0.73 to 0.22)
		ARE	5.94 (0.95)	3.13 (1.50)	2.81 (2.09)	2.27 (1.97 to 2.58)	1.96 (1.57 to 2.35)		

^a^MTB = Main Target Behaviour

^b^CSS = Clinician Severity Scale.

### Effectiveness: change in primary and secondary outcomes at pre-post and 3- and 6-month follow-ups in both treatment conditions

Means, standard deviations, and within-group and between-group effect sizes are shown in Table 2 for the ITT analysis of all the outcome measures at pre-treatment, post-treatment and 6-month follow-up. Data from 3-month follow-up are available as S1 Table.

#### Primary outcome measures

**Fig 6:**
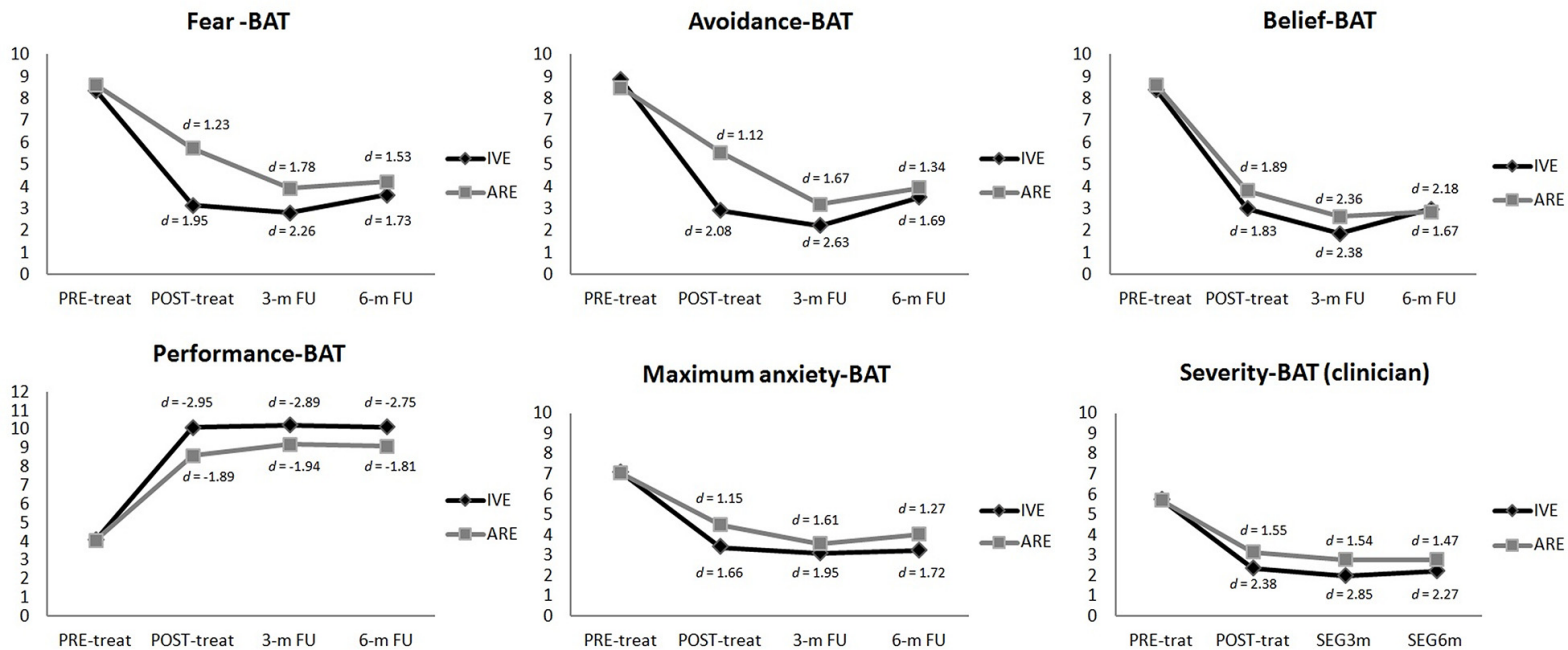
Evolution of the measures related with the primary outcome measure (the BAT) in both treatment conditions.

**ITT analysis** showed a significant time effect on all of the measures related to the **BAT**: Fear-BAT (*F*_3, 61_ = 58.73, *p* < .000); Avoidance-BAT (*F*_3, 61_ = 65.59, *p* < .000); Belief-BAT (*F*_3, 61_ = 86.71, *p* < .000); Performance-BAT (*F*_3, 61_ = 209.61, *p* < .000); maximum anxiety-BAT (*F*_3, 61_ = 45.49, *p* < .000); and Severity-BAT assessed by the clinician (*F*_3, 60_ = 89.14, *p* < .000). A significant time X treatment group interaction effect was found for the variable “Avoidance-BAT” (*F*_3, 61_ = 65.59, *p* < .020), with this interaction favoring the participants in the IVE condition. Pairwise comparisons revealed that these differences between the two conditions on this variable took place at post-treatment, while both conditions showed similar scores at the 3- and 6-month follow-ups. Fig 6 shows the evolution of the primary outcome measures at post-treatment and follows-ups as well as the effect sizes reached in each treatment condition.

**Completer analysis** also revealed a significant time effect on all of the variables related to the **BAT**, similar to the ITT analysis. However, regarding differences between the two groups, a significant time X treatment group interaction was found for the variable “Fear-BAT” (*F*_3, 45_ = 3.42, *p* < .029), with participants from the IVE group showing more improvement on this variable. In the pairwise comparisons, results revealed that this difference was present only at post-treatment, disappearing at the 3- and 6-month follow-ups, as described in the ITT analysis.

#### Secondary outcome measures

Regarding the fear related to spiders and cockroaches measured by the **FSQ**, the **ITT analysis** showed a significant time effect (*F*_3, 61_ = 158.93, *p* < .000), whereas no statistical differences were found between the two treatment conditions. Both groups improved in a similar way. In the case of beliefs related to spiders and cockroaches measured by the first scale of the SBQ (SBQ-1), a significant time effect was observed (*F*_3, 61_ = 207.70, *p* < .000), with no differences between the two conditions in the time X treatment group interaction. The same pattern was observed on the other scale of the SBQ (SBQ-2), corresponding to the participants’ beliefs about their reactions in the presence of a spider or cockroach. In this case, a significant time effect was also detected (*F*_3, 61_ = 136.13, *p* < .000), and no differences were found between the two conditions at any of the assessment moments. ITT analysis also showed a significant time effect for all of the measures related to the **MTB**: Fear-MTB (*F*_3, 61_ = 143.29, *p* < .000); Avoidance-MTB (*F*_3, 61_ = 163.31, *p* < .000); and degree of Belief-MTB related to the catastrophic thought (*F*_3, 61_ = 135.94, *p* < .000). A significant time X treatment group interaction effect was found for the variable “Avoidance-MTB” (*F*_3, 61_ = 3.20, *p* < .044), with participants from the IVE condition showing more improvement on this measure. Pairwise comparisons revealed that this difference was observed only at post-treatment, as both conditions had similar scores at the 3- and 6-month follow-ups. Regarding the last measure, the severity assessed by the clinician (**CSS**), statistical analysis showed a significant time effect in both conditions (*F*_3, 59_ = 143.29, *p* < .000), and no differences were found in terms of an interaction effect between the two conditions.

Completer analysis showed the same outcome pattern as the ITT analysis related to the time effect on these measures. Both conditions showed a significant time effect on the FSQ, SBQ, MTB measures and CSS. Regarding the time X treatment group interaction, in this analytical procedure, no differences were found on any of these measured variables, and so both conditions were equally efficacious at post-treatment and at the follow-ups.

### Effect sizes

Table 2 shows within-group and between-group effect sizes measured by Cohen’s *d* [69] for all measures obtained from the ITT analysis. As Table 2 reveals, all the measures included in the assessment reached *d* values above 0.8 in both conditions at post-treatment and follow-ups. According to Cohen, values equal to 0.8 or higher are considered large effect sizes. As for the effect sizes of the time X treatment group interaction, medium-large effect sizes, according to Cohen, were found at post-treatment for the variables: “previous avoidance (*d* = -0.80) experienced before the BAT performance” and “avoidance of the MTB” (*d* = -0.65). However, all of these variables obtained values around 0.30 or less in the time x condition interaction at follow-ups.

Taking into account the effect sizes reported in the **completer analysis**, scores obtained in the time effect analysis showed a similar pattern to the ITT analysis. As for the effect sizes of the time X treatment group interaction at post-treatment, large effect sizes, according to Cohen, were found in the variable: “previous fear (*d* = -1.06) experienced before the BAT performance”. As the ITT analysis shows, the effect size of this variable at follow-ups was *d* < 0.30.

### Duration of Exposure, Diagnostic Status, Meaningful Clinical Improvement

The analysis carried out on the **Duration of the Exposure** showed that the mean time for the IVE condition was 137 minutes (ranging from 62 to 180), and 141.83 minutes for the ARE (ranging from 70 to 180). *t*- tests revealed no differences between the two treatment conditions in this variable.

Regarding the **Diagnostic Status**, the ADIS-IV interview at post-treatment revealed a high percentage of participants with SP (cockroaches or spiders) who were diagnosis-free (IVE = 22, 71.0%; ARE = 18, 56.3%). The same thing occurred at the 3-month (IVE = 24, 77.4%; ARE = 22, 68.8%) and 6-month follow-ups (IVE = 20, 64.5%; ARE = 20, 62.5%). Regarding the statistically significant differences in these percentages, the Chi-Square test revealed no differences between the two treatment conditions at any of the assessment times.

Finally, **clinically meaningful improvement** was calculated for FSQ and SBQ scores using Jacobson and Truax’s indexes [70]. Percentages of participants from each condition who were recovered, improved, had no change or were impaired, according to these measures are available as supporting information; see S2 Table on the FSQ and SBQ Scores at Post-treatment, 3- and 6-month follow-up. As data reveal, regarding the scores for fear assessed by the FSQ and the scores for beliefs related to the feared animal (SBQ-1) and the self- reaction (SBQ-2), the percentages of participants classified as “Recovered” and “Improved” were very high in both treatment conditions at post-treatment, and also at the 3- and 6-month follow-ups. No statistically significant differences were found between the two treatment conditions in these percentages on the FSQ or the SBQ.

### Acceptance of the exposure component

Participants’ expectations and satisfaction with the treatment revealed that participants in both treatment conditions evaluated the exposure component quite positively. Means and SD obtained for expectations and satisfaction, as well as the results derived from the comparisons of the two treatment conditions, are shown as supporting information; see S3 Table.

Furthermore, the results obtained showed statistical differences between the two treatment conditions regarding expectations for the treatment: participants in ARE condition considered that the exposure component would be less “aversive” than participants of the IVE condition before treatment.

Regarding the satisfaction reported by participants after the treatment, there were significant statistical differences between the two treatment conditions: participants in IVE considered the treatment received more “useful for their problem” than participants in ARE conditions; in contrast, participants in ARE conditions found the treatment received less “aversive” than participants in IVE condition.

## Discussion

The main objective of this study was to provide empirical evidence about the efficacy and acceptance of the implementation of an AR exposure component in the treatment of small animal phobia (cockroaches and spiders) using a RCT with two conditions, IVE and ARE. The interventions in both conditions were based on the OST protocol [44]. Data obtained showed that both conditions resulted in statistically significant improvements on both primary (BAT) and secondary (FSQ, SBQ, MTB and CSS) outcome measures using both analytical procedures (ITT and completer). These results were found at post-treatment, and they were maintained at the follow-ups, obtaining large within-group effect sizes on all of the variables of more than 0.8, based on Cohen [69].

We compared ARE and IVE treatment conditions, and significant differences between the two groups were found on the primary outcome variable “Avoidance-BAT” as well as on the secondary outcome variable “Avoidance-MTB”, according to the ITT analysis, whereas the completers analysis procedure revealed differences only in the primary outcome variable “Fear-BAT”. These differences favored the IVE condition only at post-treatment, with medium-large effect sizes, but at the 3- and 6-month follow-ups, these differences were not observed. In fact, small effect sizes were obtained at follow-ups, lower than those reported by the meta-analyses carried out [28,29] (*d* = 0.35).

It is also important that no differences were found in the duration of the exposure session between the two treatment conditions.

Regarding the percentages of diagnostic status and clinically significant change estimations, results indicated significant improvements in both groups, with no significant differences between the two treatment conditions. Moreover, in both conditions, scores obtained on both the FSQ and SBQ in the last follow-up (6-month) were similar to those reported by Muris and Merckelbach [56] in a normal population and by Arntz et al. [58] in a clinical population after treatment.

In sum, both conditions (ARE and IVE) resulted in statistically significant changes in both primary and secondary outcomes, and these results were maintained at the follow-ups; additionally, no significant differences were found between the two conditions in the long term. As for the dropouts, none of the participants dropped out during the treatment, and so there were no differences between the two groups in this sense.

The results agree with those obtained in previous studies by our research group, where this AR system to apply the exposure component in cockroach phobia [43,50] obtained similar scores on both the primary outcome measure (BAT) and the secondary measures, such as the FSQ, FSQ, SBQ, MTB and CSS. In the last years, other AR systems have been developed for the treatment of SP, specifically for butterfly phobia [73] and spider phobia [74]. However, data about the efficacy of these AR systems have not been reported yet. We hope that in the near future these systems are tested and can provide additional data.

Similarly, data from the present study also coincide with findings from studies where VR was used to treat SP (animal subtype), such as those by [31, 33–35,75,76]; where VR was effective in reducing the fear and avoidance experienced by the participants. In addition, the data obtained support the meta-analysis carried out by [77], where in vivo exposure compared to other forms of SP treatment (including the use of VR) was superior at post-treatment, but not at the follow-ups. Thus, the advantage of IVE also disappeared in the long term, as occurred in the present study. We would like to have had enough statistical power to analyze whether both conditions were equally effective using an equivalence testing procedure; unfortunately, our sample size did not allow us to use this methodology. We can state that both treatment conditions were effective for the treatment of SP, with large effect sizes, but we cannot conclude that both conditions were equally efficacious.

Finally, the results obtained also agree with those from studies where the OST was applied in a traditional format, both individually (e.g. [78,46,54]) and in a group (e.g. [47,79]). The Öst one-session treatment is designed to expose the patient to the phobic situation in a planned, graded and controlled way. Moreover, it is presented to the patient as a team situation because the patient and therapist work together. One important strength of this procedure, based on the habituation rationale paradigm, is that it uses a long period of time (up to 3 hours), and this enables the patient to confront a large number of situations and test several irrational beliefs. Another advantage, according to Öst [44] and Zlomke and Davis [80], is the intensive exposure. It provides a context where “overlearning” can occur, maximizing the exposure through the generation of exposure situations beyond those often experienced in the natural environment, in order to increase exposure efficacy. However, this would be much less important in the inhibitory learning paradigm recently defended by Craske et al.[81], From this point of view, it would be much more important to appeal to strategies such as expectancy violation or variability, rather than to long exposure periods. Moreover, this intensive exposure can be quite tiring for patients and the therapist. It requires tolerating moderate distress during a prolonged session, and so patient motivation is crucial [44]. Otherwise, taking into account what was previously been mentioned about other patients suffering from phobias who do not follow any therapy, the one-session exposure would be a real advantage increasing the likelihood of receiving treatment since it is a single session. Finally, it is also necessary to state that the one-session treatment is should be a starting point, and that the patient must continue the exposure in his/her life after therapy [82].

In short, these results support the use of AR as an effective tool for the treatment of specific phobias.

Regarding the acceptance of the treatment, results obtained showed that participants from both conditions reported high expectations about the treatment before receiving it, as well as high satisfaction with it when it was applied. Significant differences were found between the two conditions: participants in IVE considered the treatment more useful for their problem at post-treatment than participants in the ARE condition; but participants in ARE considered the treatment less aversive than participants in the IVE condition, both regarding their expectations before receiving it and the satisfaction expressed after it.

This study also provides data about its applicability, as none of the participants dropped out during the treatment. These data are lower than those reported by other authors [82,83]. It is important to point out that although none of the participants refused to participate in the study, it was observed that participants from IVE condition were more reluctant to receive the treatment than participant from ARE condition. This percentage was even lower than the one reported by Öst [82] where only 0.8% of the 500 participants examined refused to participate in the different ÖST studies.

The acceptance data obtained in this study are in line with previous studies in the literature about the use of AR for the treatment of specific phobia in adults [43] and children [84]; and with studies where the VR was used to treat this problem [24,37,85,86]. In addition, as Botella et al. [50] reported, AR has some advantages, such as greater control of the exposure by the therapist (e.g. number and size of the animals, animals’ behavior, etc.), not having to keep animals in the clinic, and the possibility of interacting with a virtual element in the real world by using one’s body (e.g. placing cockroaches/spiders on hands, feet, etc.). Therefore, it is important to highlight that AR is an alternative treatment for patients and therapists, depending on their preferences.

Moreover, in recent studies, VR environments were used in the treatment of spider phobia to examine whether exposure to the phobic stimulus in different contexts and/or with different stimuli reduces the recurrence of fear [75,76,87]. Results showed that exposure to multiple virtual contexts and multiple virtual phobic stimuli reduced the recurrence of fear to a greater extent than exposure to only one scenario and the same stimuli, making it possible to generalize the results. These studies are in line with the work carried out by Craske et al. [81], focused on optimizing exposure therapy based on the inhibitory learning approach, because VR/AR can maximize some strategies reported by Craske. Some examples would be “deepened extinction”, where multiple fear stimuli (e.g. different types of spiders) are first extinguished separately before being combined during extinction (e.g. exposure to these types of spiders at the same time); “variability” (varying stimuli, durations, levels of intensity, or varying the order of the hierarchy items) because VR/AR allows greater control by therapists; and finally, “exposure to multiple contexts”. Regarding this last strategy, as mentioned above, VR/AR facilitates the exposure to different contexts, producing a positive effect in terms of fear renewal and generalization of results [75,76,87]. This effect can often be difficult to achieve in the "real world", but it can be made easier by using AR or VR, providing the therapist with much more control. For example, AR can facilitate the exposure to a range of multiple stimuli (e.g. different kind of spiders, different sizes or number of animals, etc.), and it can be used in different contexts, such as different rooms in the clinician’s office, using different lights in these rooms, at the patient’s house, or on the street (by using mobile devices), etc. For instance, at our work place, where an AR serious game on a mobile phone is used as the application device [88], the patient was able to practice exposure to the feared stimuli in different contexts, both before the One-session VR treatment with the therapist and after the session, in order to reinforce what had been learned.

This study has some limitations such as, on the one hand, the lack of a waiting list control group. However, based on previous studies on exposure technique efficacy [28,29], we decided to compare AR with IVE, the most powerful procedure for the treatment of SP at the present time. Another limitation was the sample size, which did not allow us to use an equivalence testing procedure. We would like to have had enough statistical power to analyze whether both conditions were equally effective using an equivalence testing procedure. Unfortunately, our sample size did not allow us to use this methodology. In addition, this study was conducted in a research context. Therefore, no data about clinical settings or clinicians’ acceptance were obtained. Moreover, no diagnostic interrater reliability was applied, as the assessors were not always independent from the therapists. Finally, there were some adverse effects in some cases due to the duration of the exposure session (ranging from 62 to 180 minutes). Some participants in both conditions reported feeling tired, and some of the first participants in the ARE condition suffered from dizziness and back pain due to the AR 5DT HMD. Therefore, the AR HMD was immediately replaced by a pair of AR glasses (VR goggles, Vuzix). It should be noted that these symptoms were not severe and disappeared a few hours after exposure. In any case, the administration of the Simulator Sickness Questionnaire (SSQ) would be desirable. For future studies we recommend taking breaks during the session if it is prolonged.

Regarding future lines of research, a promising line involves improving AR systems. In fact, AR systems that do not require the use of any visual device have been developed, such as the "Therapeutic Lamp" system [89], a technology based on AR-based interactive projection. The recent emergence of less invasive and more comfortable visual devices on the market at very affordable prices (e.g. “Google Glass” or “Oculus Rift”) can facilitate the use of AR by mental healthcare professionals. Similarly, an attempt has also been made to advance in this field by combining the AR system with the use of an AR serious game running on a mobile phone [88], or the use of an AR system with children as a first step before in vivo exposure [84].

The results obtained in this study provide empirical evidence about the efficacy and participants’ acceptance of AR for the treatment of specific phobias. The use of AR provides an additional option in administering exposure treatment for specific phobias and a new alternative for both patients and therapists, depending on their preferences. Finally, new research lines can be opened up, in order to define the best strategies to enhance the exposure treatment, reduce the recurrence of fear, and improve the acceptability of exposure-based treatments. According to Kazdin [13], it is necessary to consider new therapeutic models.

## Supporting Information

S1 CONSORT Checklist CONSORT 2010 Checklist. (DOC)

S1 File Ethics committee protocol English translation. (PDF)

S2 File Ethics committee protocol original document. (PDF)

S3 File Ethical approval letter. (PDF)

S1 Table Means, standard deviations, within-group and between-group effect sizes for the ITT analysis of all outcome measures at 3-month follow-up. (DOCX)

S2 Table Clinically Meaningful Improvement on the FSQ and SBQ Scores at Post-treatment, 3- and 6-month follow-up. (DOCX)

S3 Table Expectations and satisfaction with the exposure component. (DOCX)

## References

[1] Kessler RC, Chiu WT, Demler O, Walters EE. Prevalence, severity, and comorbidity of 12-month DSM–IV disorders in the national comorbidity survey replication. Arch Gen Psychiatry. 2005 6; 62: 617–27. 15939839 10.1001/archpsyc.62.6.617 PMC2847357

[2] ESEMeD/MHEDEA 2000 Investigators. Prevalence of mental disorders in Europe: Results from the ESEMeD European Study of Epidemiology of Mental Disorders (ESEMeD) project. Acta Pyschiatrica Scandinavica. 2004; 109: 21–7. 10.1111/j.1600-0047.2004.00327.x 15128384

[3] American Psychiatric Association. Diagnostic and statistical manual of mental disorders (5th ed.). Arlington, VA: American Psychiatric Publishing; 2013.

[4] Depla MFIA, ten Have ML, van Balkom AJLM, de Graaf R. Specific fears and phobias in the general population: Results from the Netherlands Mental Health Survey and Incidence Study (NEMESIS). Soc Psychiatry Psychiatr Epidemiol. 2008; 43: 200–8. 18060338 10.1007/s00127-007-0291-z

[5] LeBeau RT, Glenn D, Liao B, Wittchen HU, Beesdo-Baum K, Ollendick T, Craske MG. Specific phobia: A review of DSM–IV specific phobia and preliminary recommendations for DSM–V. Depress Anxiety. 2010; 27:148–67. 10.1002/da.20655 20099272

[6] Curtis GC, Magee WJ, Eaton WW, Wittchen HU, Kessler RC. Specific fears and phobias: Epidemiology and classification. Br J Psychiatry. 1998; 173:212–7. 9926096

[7] American Psychiatric Association. Diagnostic and statistical manual of mental disorders (4th ed., text rev.). Washington, DC: Author; 2000.

[8] Wittchen HU, Lecrubier Y, Beesdo K, Nocon A. Relationships among anxiety disorders: patterns and implications In Nutt DJ, Ballenger JC, editors. Anxiety Disorders. Oxford: Blackwell Science; 2003 p. 25–37.

[9] Antony MM, Barlow DH. Specific phobia In Barlow DH, editor. Anxiety and its disorders: The nature and treatment of anxiety and panic (2nd ed.) New York: Guilford; 2002 p. 380–417.

[10] Nathan PE, Gorman JM. A guide to treatments that work. (3rd ed.). New York: Oxford University Press; 2007.

[11] Mackenzie CS, Reynolds K, Cairney J, Streiner D, Sareen J. Disorder-specific mental health service use for mood and anxiety disorders: Associations with age, sex, and psychiatric comorbidity. Depress Anxiety. 2012; 29(3):234–42. 10.1002/da.20911 22065571 PMC4284961

[12] Stinson FS, Dawson DS, Chou SP, Smith S, Goldstein RB, Ruan WJ, et al The epidemiology of DSM-IV specific phobia in the USA: Results from the national epidemiologic survey on alcohol and related conditions. Psychol Med. 2007; 37(7): 1047–59. 17335637 10.1017/S0033291707000086

[13] Kazdin AE. Evidence-based psychotherapies II: changes in models of treatment and treatment delivery. S Afr J Psychol. 2014. 0081246314538733.

[14] Kazdin AE, Blase SL. Rebooting psychotherapy research and practice to reduce the burden of mental illness. Perspect Psychol Sci. 2011; 6(1):21–37. 10.1177/1745691610393527 26162113

[15] Kazdin AE, Rabbitt SM. Novel models for delivering mental health services and reducing the burdens of mental illness. Clin Psychol Sci. 2013; 1(2):170–91.

[16] Olatunji BO, Deacon BJ, Abramowitz JS. The cruelest cure? Ethical issues in the implementation of exposure-based treatments. Cogn Behav Pract. 2009; 16(2):172–80.

[17] Moritz K, Hoffman J, Herbert JD, Schare ML. Anxiety Disorders: Navigating Legal and Ethical Dilemmas. Paper presented at the 47th Annual Convention of Cognitive and Behavioral Therapies (ABCT2013), Nashville. 2013, Nov. Available: http://abct2013.abstractcentral.com.

[18] Richard DCS, Gloster AT. Exposure therapy has a public relations problem: A dearth of litigation amid a wealth of concern Comprehensive handbook of the exposure therapies. New York: Academic Press; 2007 p. 409–25.

[19] Wolitzky-Taylor KB, Viar-Paxton MA, Olatunji BO. Ethical Issues When Considering Exposure In Davis T.E, Ollendick TH, Öst LG, editors. Intensive One-Session Treatment of Specific Phobias. New York: Springer; 2012 p. 195–208.

[20] Becker CB, Zayfert C, Anderson E. A survey of psychologists’ attitudes towards and utilization of exposure therapy for PTSD. Behav Res Ther. 2004; 42(3): 277–92. 14975770 10.1016/S0005-7967(03)00138-4

[21] Hembree EA, Foa EB, Dorfan NM, Street GP, Kowalski J, Tu X. Do patients drop out prematurely from exposure therapy for PTSD?. J Trauma Stress. 2003; 16(6): 555–62. 14690352 10.1023/B:JOTS.0000004078.93012.7d

[22] Abramowitz JS. The practice of exposure therapy: relevance of cognitive-behavioral theory and extinction theory. Behav Ther. 2013; 44(4): 548–58. 10.1016/j.beth.2013.03.003 24094780

[23] Choy Y, Fyer AJ, Lipsitz JD. Treatment of specific phobia in adults. Clin Psychol Rev. 2007; 27(3): 266–86. 17112646 10.1016/j.cpr.2006.10.002

[24] Garcia-Palacios A, Botella C, Hoffman H, Fabregat S. Comparing acceptance and refusal rates of virtual reality exposure vs. in vivo exposure by patients with specific phobias. Cyberpsychol Behav. 2007; 10(5): 722–4. 17927544 10.1089/cpb.2007.9962

[25] Konnopka A, Leichsenring F, Leibing E, König HH. Cost-of-illness studies and cost-effectiveness analyses in anxiety disorders: a systematic review. J Affect Disorders. 2009; 114(1), 14–31. 18768222 10.1016/j.jad.2008.07.014

[26] McCann RA, Armstrong CM, Skopp NA, Edwards-Stewart A, Smolenski DJ, June JD, et al Virtual reality exposure therapy for the treatment of anxiety disorders: An evaluation of research quality. J Anxiety Disord. 2014; 28(6): 625–31. 10.1016/j.janxdis.2014.05.010 25093964

[27] Meyerbröker K, Emmelkamp PM. Virtual reality exposure therapy in anxiety disorders: a systematic review of process-and-outcome studies. Depress Anxiety. 2010; 27(10): 933–44. 10.1002/da.20734 20734361

[28] Opriş D, Pintea S, García-Palacios A, Botella C, Szamosközi Ş, David D. Virtual reality exposure therapy in anxiety disorders: a quantitative meta-analysis. Depress Anxiety. 2012; 29(2): 85–93. 10.1002/da.20910 22065564

[29] Powers MB, Emmelkamp PM. Virtual reality exposure therapy for anxiety disorders: A meta-analysis. J Anxiety Disord. 2008; 22(3): 561–9. 17544252 10.1016/j.janxdis.2007.04.006

[30] Turner WA, Casey LM. Outcomes associated with virtual reality in psychological interventions: where are we now? Clin Psychol Rev. 2014; 8(34): 634–44. 10.1016/j.cpr.2014.10.003 25455627

[31] Bouchard S, Côté S, St-Jacques J, Robillard G, Renaud P. Effectiveness of virtual reality exposure in the treatment of arachnophobia using 3D games. Techno Health Care. 2006; 14(1):19–27. 16556961

[32] Bouchard S, St-Jacques J, Robillard G, Renaud P. Efficacité d’un traitement d’exposition en réalité virtuelle pour le traitement de l’arachnophobie chez l’enfant une étude pilote. Journal de thérapie comportementale et cognitive. 2003; 17(3): 101–8.

[33] Garcia-Palacios A, Hoffman H, Carlin A, Furness TA, Botella C. Virtual reality in the treatment of spider phobia: a controlled study. Behav Res Ther. 2002; 40(9), 983–93. 12296495 10.1016/s0005-7967(01)00068-7

[34] Hoffman HG, García-Palacios A, Carlin A, Furness TA, Botella C. Interfaces that heal: coupling real and virtual objects to treat spider phobia. Int J Hum Comput Int. 2003; 16(2): 283–300.

[35] Michaliszyn D, Marchand A, Bouchard S, Martel MO, Poirier-Bisson J. A randomized, controlled clinical trial of in virtuo and in vivo exposure for spider phobia. Cyberpsychol Behav Soc Netw. 2010; 13(6): 689–95. 10.1089/cyber.2009.0277 21142994

[36] Quero S, Pérez-Ara MA, Bretón-López J, García-Palacios A, Baños RM, Botella C. Acceptability of virtual reality interoceptive exposure for the treatment of panic disorder with agoraphobia. Br J Guid Counc. 2014; 42(2):123–37. 20543274

[37] Rothbaum BO, Anderson P, Zimand E, Hodges L, Lang D, Wilson J. Virtual reality exposure therapy and standard (in vivo) exposure therapy in the treatment of fear of flying. Behav Ther. 2006; 37(1): 80–90. 16942963 10.1016/j.beth.2005.04.004

[38] Sarver NW, Beidel DC, Spitalnick JS. The Feasibility and Acceptability of Virtual Environments in the Treatment of Childhood Social Anxiety Disorder. J Clin Child Adol Psychol. 2014; 43(1), 63–73. 10.1080/15374416.2013.843461 PMC3947271 24144182

[39] Azuma RT. A survey of augmented reality. Presence. 1997; 6(4): 355–85.

[40] Dunleavy M, Dede C. Augmented reality teaching and learning In: Spector JM, Merrill MD, Elen J, Bishop MJ, editors. Handbook of research on educational communications and technology. New York: Springer; 2014 p. 735–45.

[41] Zhu E, Hadadgar A, Masiello I, Zary N. Augmented reality in healthcare education: an integrative review. 2014; (No. e335v2). PeerJ PrePrints. 10.7717/peerj.469 PMC4103088 25071992

[42] Quero S, Pérez-Ara MA, Campos D, Cortés-Díaz S, Collado EJ, Baños RM, et al Preliminary data of Augmented Reality Training Program for Safety at work in hospitals In: Gómez L, López A, Candel I, editors. ICERI2014: Proceedings of the 7th International Technology, Education, and Development Conference; 2014 nov 17–19; Sevilla, Spain IATED Academy; 2014 p. 1568–73.

[43] Botella CM, Juan MC, Baños RM, Alcañiz M, Guillén V, Rey B. Mixing realities? An application of augmented reality for the treatment of cockroach phobia. Cyberpsychol Behav: the impact of the Internet, multimedia and virtual reality on behavior and society; 2005, 8(2): 162–71. 10.1089/cpb.2005.8.162 15938656

[44] Öst LG. One-session treatment for specific phobias. Behav ResTher. 1989; 27(1): 1–7. 10.1016/0005-7967(89)90113-7 2914000

[45] Öst LG. Rapid treatments of specific phobias In: Davey GCL, editor. Phobias. A handbook of theory, research and treatment. Chichester: John Wiley and Sons; 1997 p. 227–46.

[46] Koch EI, Spates CR, Himle JA. Comparison of behavioral and cognitive-behavioral one-session exposure treatments for small animal phobias. Behav Res Ther. 2004; 42(12): 1483–1504. 15500817 10.1016/j.brat.2003.10.005

[47] Götestam KG. One session group treatment of spider phobia by direct or modelled exposure. Cogn Behav Ther. 2002; 31(1): 18–24.

[48] Schienle A, Schäfer A, Hermann A, Rohrmann S, Vaitl D. Symptom provocation and reduction in patients suffering from spider phobia. Eur Arch Psychiatry Clin Neurosci. 2007; 257(8), 486–93. 17902000 10.1007/s00406-007-0754-y

[49] Bretón-López J, Quero S, Botella C, García-Palacios A, Baños RM, Alcañiz M. An augmented reality system validation for the treatment of cockroach phobia. Cyberpsychol Behav Soc Netw. 2010; 13(6): 705–10. 10.1089/cyber.2009.0170 21142997

[50] Botella C, Bretón-Lopez J, Quero S, Baños RM, García-Palacios A. Treating Cockroach Phobia with augmented reality. Behav Ther. 2010; 41(3): 401–13. 10.1016/j.beth.2009.07.002 20569788

[51] Moher D, Schulz KF, Altman DG, Group C. The CONSORT statement: revised recommendations for improving the quality of reports of parallel-group randomized trials. J Am Podiatr Med Assoc. 2001; 91(8), 437–42. 11574648 10.7547/87507315-91-8-437

[52] Moher D, Hopewell S, Schulz KF, Montori V, Gotzsche PC, Devereaux PJ, et al CONSORT 2010 Explanation and Elaboration: Updated guidelines for reporting parallel group randomised trials. J Clin Epidemiol. 2010; 63(8): e1–37. 10.1016/j.jclinepi.2010.03.004 20346624

[53] Eysenbach G, Group CE. CONSORT-EHEALTH: improving and standardizing evaluation reports of Web-based and mobile health interventions. J Med Internet Res. 2011; 13(4): e126 10.2196/jmir.1923 22209829 PMC3278112

[54] Öst LG, Salkovskis PM, Hellström K. One-session therapist-directed exposure vs. self-exposure in the treatment of spider phobia. Behav Ther. 1991; 22(3): 407–22. 10.1016/0005-7967(95)00028-v 7487856

[55] Szymanski J, O'Donohue W. Fear of spiders’ questionnaire. J Behav Ther Exp Psychiatry. 1995; 26(1): 31–4. 7642758 10.1016/0005-7916(94)00072-t

[56] Muris P, Merckelbach H. A comparison of two spider fear questionnaires. J Behav Ther Exp Psychiatry. 1996; 27(3): 241–4. 8959425 10.1016/s0005-7916(96)00022-5

[57] Nebot S, Quero S, Bretón-López J, Pérez-Ara MA, Botella C. Validación Española del Cuestionario de Miedo a las Arañas (FSQ) adaptado para la Fobia a las Cucarachas. SEAS2012: Poster presentation at the IX International Conference of the Sociedad Española para el estudio de la Ansiedad y Estrés; 2012 Sep 6–8; Valencia, Spain.

[58] Arntz A, Lavy E, van den Berg G, van Rijsoort S. Negative beliefs of spider phobics: A psychometric evaluation of the spider phobia beliefs questionnaire. Adv Behav Res Ther. 1993; 15(4): 257–77.

[59] Nebot S, Quero S, Pérez-Ara MA, Bretón-López J, Molés M, Rachila I, et al. Validación Española del Cuestionario de Creencias de Fobia a las Arañas adaptado para la fobia a las cucarachas en población general y clínica”. SEAS2013: Poster presentation at the XX anual meeting of the Sociedad Española para el estudio de la Ansiedad y Estrés (SEAS); 2013 oct 3–4; Madrid, Spain.

[60] Marks IM, Mathews AM. Brief standard self-rating for phobic patients. Behav Res Ther. 1979; 17: 263–7. 526242 10.1016/0005-7967(79)90041-x

[61] Di Nardo P, Brown TA, Barlow DH. Anxiety Disorders Interview Schedule for DSM–IV (Lifetime Version). San Antonio, TX: The Psychological Corporation; 1994.

[62] Antony MM, Orsillo SM, Roemer L. Practitioners’s guide to empirically based measures of anxiety. New York, N.Y.: Kluwer Academic/Plenum Publishers; 2001.

[63] Di Nardo PA, Moras K, Barlow DH, Rapee RM, Brown TA. Reliability of DSM–III–R anxiety disorder categories: Using the Anxiety Disorders Interview Schedule–Revised (ADIS–R). Arch Gen Psychiatry. 1993; 50: 251–6. 8466385 10.1001/archpsyc.1993.01820160009001

[64] Öst LG, Stridh BM, Wolf M. A clinical study of spider phobia: Prediction of outcome after self-help and therapist-directed treatments. Behav Res Ther. 1998; 36(1): 17–35. 9613014 10.1016/s0005-7967(97)10018-3

[65] Borkovec TD, Nau SD. Credibility of analogue therapy rationales. J Behav Ther Exp Psy. 1972; 3(4): 257–60.

[66] Botella C, García‐Palacios A, Villa H, Baños RM, Quero S, Alcañiz M, et al Virtual reality exposure in the treatment of panic disorder and agoraphobia: A controlled study. Clin Psychol Psychot. 2007; 14(3): 164–75.

[67] Baños RM, Botella C, Guillen V, García-Palacios A, Quero S, Bretón-López J, et al An adaptive display to treat stress-related disorders: EMMA's World. Brit J Guid Couns. 2009; 37(3): 347–56.

[68] Schwartz D, Lellouch J. (1967). Explanatory and pragmatic attitudes in therapeutical trials. J Chron Dis. 1967; 20(8): 637–48. 4860352 10.1016/0021-9681(67)90041-0

[69] Cohen J. Statistical Power Analysis for the Behavioral Sciences (2nd ed.). Hillsdale, NJ: Erlbaum; 1988.

[70] Jacobson NS, Truax P. Clinical significance: a statistical approach to defining meaningful change in psychotherapy research. Journal of consulting and clinical psychology. 1991; 59(1): 12 2002127 10.1037//0022-006x.59.1.12

[71] Iraurgi I. Evaluación de resultados clínicos (y III): Índices de Cambio Fiable (ICF) como estimadores del cambio clínicamente significativo. Norte de Salud mental. 2010; 8(36).

[72] Kupfer DJ. Long-term treatment of depression. J Clin Psychiatry. 1991; 52, 28–34. 1903134

[73] Abate AF, Nappi M, Ricciardi S. AR based environment for exposure therapy to mottephobia In: Shumaker R, editors. Virtual and Mixed Reality- New Trends, Part I. Springer Berlin Heidelberg; 2011 p. 3–11.

[74] Corbett-Davies S, Dünser A, Clark A. An Interactive Augmented Reality System for Exposure Treatment Proceeding of the 11th IEEE International Symposium on Mixed and Augmented Reality (ISMAR); 2012 11 5–8; Atlanta, Georgia, USA Available: http://www.hitlabnz.org.

[75] Shiban Y, Pauli P, Mühlberger A. Effect of multiple context exposure on renewal in spider phobia. Behav Res Ther. 2013; 51(2): 68–74. 10.1016/j.brat.2012.10.007 23261707

[76] Shiban Y, Schelhorn I, Pauli P, Mühlberger A. Effect of combined multiple contexts and multiple stimuli exposure in spider phobia: A randomized clinical rial in virtual reality. Behav Res Ther. 2015; 71: 45–53. 10.1016/j.brat.2015.05.014 26072451

[77] Wolitzky-Taylor KB, Horowitz JD, Powers MB, Telch MJ. Psychological approaches in the treatment of specific phobias: A meta-analysis. Clin Psychol Rev. 2008, 28(6):1021–37. 10.1016/j.cpr.2008.02.007 18410984

[78] Hellström K, Öst LG. One-session therapist directed exposure vs. two forms of manual directed self-exposure in the treatment of spider phobia. Behav ResTher. 1995, 33(8): 959–65. 10.1016/0005-7967(95)00028-v 7487856

[79] Leutgeb V, Schäfer A, Schienle A. An event-related potential study on exposure therapy for patients suffering from spider phobia. Biol Psychol. 2009; 82(3): 293–300. 10.1016/j.biopsycho.2009.09.003 19751797

[80] Zlomke K, Davis TE III. One-session treatment of specific phobias: A detailed description and review of treatment efficacy. Behav Ther. 2008; 39(3): 207–23. 10.1016/j.beth.2007.07.003 18721635

[81] Craske MG, Treanor M, Conway CC, Zbozinek T, Vervliet B. Maximizing exposure therapy: An inhibitory learning approach. Behav Res Ther. 2014; 58, 10–23. 10.1016/j.brat.2014.04.006 24864005 PMC4114726

[82] Öst LG. One-Session Treatment: Principles and Procedures with Adults In: Davis T III, Ollendick T, Öst L, editors. Intensive One-Session Treatment of Specific Phobias. London: Springer; 2012 p. 59–95.

[83] Garcia-Palacios A, Hoffman HG, Kwong See S, Tsai A, Botella C. Redefining therapeutic success with virtual reality exposure therapy. CyberPsychol Behav. 2001; 4(3): 341–8. 11710258 10.1089/109493101300210231

[84] Quero S, Nebot S, Rasal P, Bretón-López J, Baños RM, Botella C. Las tecnologías de la información y la comunicación en el tratamiento de la fobia a los animales pequeños en la infancia. Behav Psychol. 2014; 22(2).

[85] Tortella-Feliu M, Botella C, Llabrés J, Bretón-López JM, Riera del Amo A. Baños RM, et al Virtual reality versus computer-aided exposure treatments for fear of flying. Behav Modif. 2011; 35(1): 3–30. 10.1177/0145445510390801 21177516

[86] Botella C, Quero S, Baños RM, Garcia-Palacios A, Breton-Lopez J, Alcaniz M, et al Telepsychology and self-help: the treatment of phobias using the internet. Cyberpsychol Behav. 2008; 11(6): 659–64. 10.1089/cpb.2008.0012 18991528

[87] Dunsmoor JEF, Zielinski DJ, LaBar KS. Extinction in multiple virtual reality contexts diminishes fear reinstatement in humans. Neurobiol Learn Mem. 2014; 113: 157–64. 10.1016/j.nlm.2014.02.010 24583374 PMC4053498

[88] Botella C, Breton-López J, Quero S, Baños RM, García-Palacios A, Zaragoza I, et al Treating cockroach phobia using a serious game on a mobile phone and augmented reality exposure: A single case study. Comput Hum Behav. 2011; 27(1): 217–27.

[89] Wrzesien M, Raya MA, Botella C, Burkhardt JM, Bretón-López J, Ortega M, et al The Therapeutic Lamp: Treating Small-Animal Phobias. IEEE Comput Graph. 2013; 33(1): 80–6. 10.1109/MCG.2013.12 24807885

