# Supplementary material for: In Vivo versus Augmented Reality Exposure in the Treatment of Small Animal Phobia: A Randomized Controlled Trial
Source: PLoS One. 2016 Feb 17;11(2):e0148237. doi: 10.1371/journal.pone.0148237 (PMC4757089; doi:10.1371/journal.pone.0148237)
Supplement: S1 File — (PDF) [file pone.0148237.s002.pdf]

Research report for the application for approval of the Ethics Committee of the Universitat Jaume I for the Project entitled:

"The efficacy of the use of the in vivo exposure versus the Augmented Reality exposure for the treatment of the phobia to small animals".

LabPsitec

The Psychology and Technology Lab (LabPsiTec) of the Universitat Jaume I of Castellón and Universitat de Valencia is a research group with more than 15 years of experience researching the possibilities the Information and Communication Technologies (ICTs) may offer to Clinical Psychology as assessment and treatment tools.

Nowadays, the group has a set of applications that have proven its efficacy for the treatment of phobias (i.e., acrophobia, spider phobia, cockroach phobia, fear to talk, claustrophobia, fear to fly), panic disorder with and without agoraphobia, post-traumatic stress disorder, pathological grief, eating disorders and obesity, pathological gambling, etc. Specifically, the treatment to the fear to small animals through the use of the Augmented Reality system (AR) is an original and recent research line within the field of new technologies and Clinical Psychology.

AR involves adding virtual elements to the real world. We have designed a program to recreate virtual elements for the treatment to phobia to small animals, specifically for spiders and cockroaches. During the treatment with the therapist and within a real context (a table, the floor, the patient's personal objects, etc…) cockroaches or virtual spiders are reproduced. These stimuli may vary in number, the place they show up, size or state (static or in movement, dead or alive) so that the patient can be exposed to each of the scenarios. The experience within this field indicates that virtual objects (in this case, small animals) are highly valued by the participants, as they are immersed in a real environment.

The study to be carried out intends to test, in a controlled study, the efficacy and efficiency of the AR system as a therapeutic tool to treat the phobia to small animals.

UNIVERSITAT JAUME•I

Introduction

Specific phobias are the most prevalent ones among anxiety disorders. The overall prevalence rate is quite high (around 7.2% and 11.3% among the general population). Fear to cockroaches or spiders is a quite specific phobia classified within the phobia to animals subtype (APA, 2000). These phobias are described as a marked, persistent, excessive, and irrational fear. Exposure to these animals causes in a virtually steady way an immediate anxiety response, even though the person admits his fear to be excessive and irrational (APA, 2000). As a consequence, the phobic situation is avoided or else the person faces it experiencing intense discomfort, which provokes high levels of interference in the person's life (family circle, professional, social activities, etc…).

Nowadays, the treatment of choice for specific phobias (including the phobia to small animals), is the in vivo exposure. That is to say, a therapist gradually introduces those feared stimuli to the patient. This way, the difficulty of the situations the patient is exposed to is being increased, from low to higher anxiety (Agras, Sylvester & Oliveau, 1969; Boyd et al., 1990; Essau, Conrad, and Petermann, 2000; Magee, Eaton, Wittchen, McGonagle and Kessler, 1996). Also, not all patients benefit from the in vivo exposure treatment, as among all those who seek treatment, a high percentage either reject it or drop out (around a 25%) once they are informed of the procedure that is going to be followed when applying the in vivo exposure (García-Palacios, Botella, Hoffman and Fabregat, 2007; García-Palacios, Hoffman, See, Tsay and Botella, 2001; Marks, 1978, 1992).

Information and Communication Technologies, such as Virtual Reality (VR) or Augmented Reality (AR) are pioneer applications which may improve to a great extent the adherence to the treatment and therefore, its acceptance (García-Palacios, Botella, Hoffman and Fabregat, 2007). Patients can then interact with virtual environments related to their fears, in a way that guarantees the exposure in a safe and more controlled context, with the support of the therapist.

So far there are a number of studies which offer preliminary data regarding the utility of the new technologies, specifically the use of the AR for the treatment of the phobia to small animals (Botella et al., 2005; Botella, Bretón-López, Quero, Baños & García-Palacios, 2010; Juan et al., 2005). These studies have proven that the exposure through AR may achieve an important decrease of fear, avoidance, and the beliefs related to the situations which mean facing these phobic elements (spiders or cockroaches). The participants who have received treatment with these systems have significantly improved with the treatment and have kept those improvements along the different follow ups (Botella et al., 2010). However, so far we do not have any control study which may allow us assess the differential efficacy of the new technologies for the treatment of the phobia to small animals versus the in vivo traditional therapy (that is, versus the treatment of choice at the time being). That is why, our goal here is to study, through a control study, the differential efficacy of both treatment options as well as the preferences showed by the participants, once the treatment is over. The hypothesis set in our study is that there would not be statistically significant differences regarding efficacy between the in vivo exposure treatment and the treatment through Augmented Reality for the phobia to small animals. However, there would be statistically significant differences regarding efficiency seen as the preferences of the participants of either one or another treatment.

Methodology

Study design and sample: A between-groups design will be conducted. The sample size will be at least 50 participants, 25 randomized assigned to each experimental condition (in vivo exposure and exposure through AR).

Inclusion criteria: To be included in the study the following criteria are considered:

1) Be aged between 18 and 60 years old.
2) Diagnosis of small animal phobia (cockroaches or spiders) according to DSM-IV-TR criteria.
3) Having minimum 1-year duration of the phobia.
4) No suffering from another psychological problem that requires immediate attention.
5) No suffering serious physical diseases.
6) No currently suffering from alcohol or drug dependency.
7) No currently being treated in a similar treatment program.
8) No taking anxiolytics during the study (or in case of taking them, changing the drug or dose during the study).
9) Being willing to participate in the study.

Study protocol

Before the assessment: Admission Interview (screening).

SESSION 1: ASSESSMENT

- Consent form of participation in the study.
- Consent form of sessions recording.
- The Anxiety Diagnostic Interview Schedule IV (ADIS-IV): Specific Phobia.
- Fear and Avoidance Scales (Adapted from Marks and Mathews, 1979).
- Expectations of change of the target behaviors.
- Clinician Severity Scale.
- Behavioral Avoidance Test.
- Expectations regarding the exposure treatment.

Questionnaires to complete at the end of the session in the therapy room:

- Adjustment scale (Echeburúa and de Corral, 1992).
- Fear of Spiders Questionnaire (FSQ; adapted from Szymanski and O'Donohue 1995).
- Spider Phobia Beliefs Questionnaire (SBQ; adapted from Arntz, Lavy, van der Berg and van Rijssoort, 1993).
- Disgust propensity and sensitivity scale (DPSS-R-12; Sandin et al., 2008).
- Beck Depression Inventory (BDI-II ; 2007 version).
- Preference questionnaire about AR or In Vivo exposure.

SESSION 2: TREATMENT

Intensive treatment session in AR or In Vivo exposure treatment condition. After the session, participants from AR exposure condition fill:

- CPYJR.
- Presence questionnaire (Slater).

Post-treatment assessment protocol for all participants:

- The Anxiety Diagnostic Interview Schedule IV (ADIS-IV): Specific Phobia.
- Fear and Avoidance Scales (Adapted from Marks and Mathews, 1979).
- Improvement Scale of the phobia (assessed by the therapist).
- Clinician Severity Scale.
- Behavioral Avoidance Test.
- Satisfaction regarding the exposure treatment received.
- Questionnaire of treatment preferences: AR versus In vivo exposure.

Questionnaires to complete at the end of the session in the therapy room:

- Adjustment scale (Echeburúa and de Corral, 1992).
- Fear of Spiders Questionnaire (FSQ; adapted from Szymanski and O'Donohue 1995).
- Spider Phobia Beliefs Questionnaire (SBQ; adapted from Arntz, Lavy, van der Berg and van Rijssoort, 1993).
- Disgust propensity and sensitivity scale (DPSS-R-12; Sandin et al., 2008).
- Beck Depression Inventory (BDI-II ; 2007 version).

Follow-ups (1, 3, 6 and 12 months):

- The Anxiety Diagnostic Interview Schedule IV (ADIS-IV): Specific Phobia.
- Fear and Avoidance Scales (Adapted from Marks and Mathews, 1979).
- Clinician Severity Scale.
- Improvement Scale of the phobia (assessed by the therapist).
- Behavioral Avoidance Test.
- Satisfaction questionnaire regarding the exposure treatment received
- Adjustment scale (Echeburúa and de Corral, 1992).
- Fear of Spiders Questionnaire (FSQ; adapted from Szymanski and O'Donohue 1995).
- Spider Phobia Beliefs Questionnaire (SBQ; adapted from Arntz, Lavy, van der Berg and van Rijssoort, 1993).
- Disgust propensity and sensitivity scale (DPSS-R-12; Sandin et al., 2008).
- Beck Depression Inventory (BDI-II ; 2007 version).
- Questionnaire of treatment preferences: AR versus In vivo exposure.

Statistical Analysis of the data

SPSS v.17 will be use to data analysis. Appropiate statistical procedures and strateges will be use for data analysis (MANOVAS, ANOVAS, effect sizes, Meaningful

Clinical Improvement analysis, etc.) in order to determine the compliance of the hypothesis proposed in the study.

Implications of the study

This study provides an in-depth and rigorous way of analysis the efficacy of the new technologies, specifically AR versus traditional treatment for a specific phobia. The comparison between the two treatments, as well as the preferences reported by participants, will provide an important advance in terms of the benefits this technology can contribute to treat those problems that cause an important interference in daily life of people.

The results are intended to provide information of the clinical utility of current technological developments in order to improve the efficacy and efficiency of the psychological evidence-based treatments. Finally, it is important to point out that so far this is the first randomized controlled trial to examine the differential efficacy and efficiency of both treatments, so this study could be an important contribution for the current literature related to these issues.

Bibliography

Agras, S., Sylvester, D., and Oliveau, D. (1969). The epidemiology of common fears and phobia. *Comprehensive Psychiatry, 10,* 151-156.

American Psychiatric Association, APA (2000). *Diagnostic and statistical manual for mental disorders (4ª edition, revised text) (DSM-IV-TR).* Washington, DC: Autor.

Botella, C., Bretón-López, J., Quero, S., Baños, R. and García-Palacios, A. (2010). Treating Cockroach Phobia With Augmented Reality. *Behavior Therapy, 41,* 401-413.

Botella, C., Juan, M.C., Baños, R., Alcañiz, M., Guillén, V., and Rey, B. (2005). Mixing realities? an application of augmented reality for the treatment of cockroach phobia. *CyberPsychology & Behavior, 8,* 162-171.

Boyd, J.H., Rae, D.S., Thompson, J.W., Burns, B.J., Bourdon, K., Locke, B.Z., et al. (1990). Phobia: prevalence and risk factors. *Social Psychiatrica and Psychiatric Epidemiology, 25,* 314-323.

Essau, C.A., Conradt, J., and Petermann, F. (2000). Frequency, comorbidity and psychosocial impairment of specific phobia in adolescents. *Journal of Clinical Child Psychology, 29,* 221-231.

García-Palacios, A., Botella, C., Hoffman, H., and Fabregat, S. (2007). Comparing acceptance and refusal rates of virtual reality exposure vs. in vivo exposure by patients with specific phobias. *CyberPsychology & Behavior, 10,* 722-724.

García-Palacios, A., Hoffman, H.G., See, S.K., Tsay, A., and Botella, C. (2001). Redefining therapeutic success with virtual reality exposure therapy. *CyberPsychology & Behavior, 4,* 341-348.

Juan, M. C., Alcañiz, M., Monserrat, C., Botella, C., Baños, R., and Guerrero, B. (2005). Using augmented reality to treat phobias. *IEEE Computer Graphics and Applications, 25,* 31-37.

Magee, W., Eaton, W.W., Wittchen, H.U., McGonagle, K.A., and Kessler, R.C. (1996). Agoraphobia, simple phobia, and social phobia in the national comorbidity survey. *Archives of General Psychiatry, 53,* 159-168.

Marks, I.M. (1978). Behavioral psychotherapy of adult neurosis. In S.L. Gardfield & A.E. Bergin (eds.), *Handbook of psychotherapy and behaviour change*, (pp. 493-598). New York: Wiley.

Marks, I.M. (1992). Tratamiento de exposición en la agorafobia y el pánico. In E. Echeburúa (ed.), *Avances en el tratamiento psicológico de los trastornos de ansiedad* (pp. 35-55). Madrid: Pirámide.

CONSENT FORM

The undersigned, Mr / Ms…………………………………………………………
has been informed regarding the following:

- Participation in this project is completely voluntary and I can abandon it at any moment, without any sort of consequences.
- The objective of this project is to try to improve the comprehension of the diagnosis and treatment of fear to small animals (spiders and cockroaches), as well as improving the diagnostic and therapeutic procedures used for these problems.
- The participation in this project poses no risk for my physical nor mental health.
- In case I feel fear to any of the mentioned animals I will be able to take part in the study centered on the application of a psychological treatment in order to help me overcome this problem.
- My right to privacy and confidentiality of the information provided will at any time be preserved.

Once I have been informed of all the above details and all the questions I may arise have been answered, I give my voluntary consent for any data resulting from my participation in this project to be published in the field of scientific dissemination.

Which I declare and sign on ……………………………….

CONSENT FORM VIDEO RECORDING/AUDIO

Name and surname: ____

One of the resources used at the Emotional Disorders Clinic at Jaume I Universitiy, is the recording of the sessions in video and/or audio. The recordings provide important information used as:

- As a therapeutic tool (e.g. at some point during the treatment, the therapist may ask the patient to watch one or more sessions as a homework task).

- To analyze and work in a specific case during the therapeutic process by the different therapists of the Emotional Disorders Clinic.

- To be used as training material at postgraduate courses (for graduates in psychology/medicine) and to be shown at specialized conferences. In that case, the images of the video recording are distorted and anything that might lead to the identification of the person is deleted.

I DECLARE:

I have been properly informed of the possible use of recordings during the sessions of the psychological treatment I will receive at the Emotional Disorders Clinic at Jaume I University.

That, always preserving my right to privacy and anonymity, I agree and voluntary accept the recording of my therapy sessions.

That I can change my mind and ask not to carry out the recording sessions at any time.

Signature of the interested person	Signature of the Head of the Emotional Disorders Clinic at Jaume I University.
Date and place	Date and place

Emotional Disorders Clinic: Data protection and ethical issues

The Emotional Disorders Clinic at Jaume I Universitiy follows the principles of the Helsinki Declaration and the Convention of the Council of Europe on Human Rights and Biomedicine. In addition, research carried out at the clinic also follows the Code of Ethics established by the Official College of Psychologists and it conforms the Data Protection Act, Organic Law 15/1999.

At the Emotional Disorders Clinic, the following measures for data protection are followed:

- There is a specific file archive to keep the clients' clinical records. This room has an electronic lock file that records accesses (person, data, and time). In addition, the clinical records are located at lockable metal file cabinets, kept by the administrative staff. All data related to the clients of the Clinic since its opening at December 1993, are kept at the file archive.

- Only three members of the Emotional Disorders Clinic have access to the file archive: the clinical coordinator, the administrative officer, and the person responsible for equipment and infrastructure. The way to access to the clinical records is to ask for the file to the administrative officer. After the file consultation, the officer returns the file to the file archive.

- Patients are informed both orally and in writing, that their personal data will be treated confidentially. Written information includes the permission request for the data collected during the evaluation and treatment to be used for research, always preserving the right to privacy and anonymity.

- Oral information is included in all studies carried out in the Emotional Disorders Clinic and in writing (consent forms) for participants; it is also clear that all research carried out at the clinic conforms the national and international ethical standards (e.g. code of ethics of the Official College of Psychologists, APA, and Declaration of Helsinki).

- All staff at the clinic is subject to the ethical code which includes safeguarding the privacy and anonymity of all patients.

- As for the computer media: the creation of databases with information from patients regarding the evaluation and treatment results, does not contain any personal data identifying the patient. Instead, each participant is assigned a numeric code. The link between the numeric code and the participant remains only on paper and is treated as a clinical record, kept at the file archive.

- As for the data management, SPSS is used for the analysis of the participants' data, by using numeric codes for the clients association. From that moment on,

therapists should work only with the numeric code. Names or information that allows the identification of clients are not shown at all.

- As for the audios and videos generated, they are stored at a locked cabinet, in an audiovisual control room with an electronic lock and access registration. The key of this cabinet is kept by the person responsible of equipment and infrastructure.
