# Supplementary material for: In Vivo versus Augmented Reality Exposure in the Treatment of Small Animal Phobia: A Randomized Controlled Trial
Source: PLoS One. 2016 Feb 17;11(2):e0148237. doi: 10.1371/journal.pone.0148237 (PMC4757089; doi:10.1371/journal.pone.0148237)
Supplement: S2 File — (PDF) [file pone.0148237.s003.pdf]

Memoria de investigación para la solicitud de aprobación de la Comisión de Ética de la Universitat Jaume I para el proyecto titulado:

“La eficacia del uso de la terapia de exposición tradicional (exposición en vivo) versus exposición por medio de Realidad Aumentada para el tratamiento de la fobia a los animales pequeños”

LabPsitec

El Laboratorio de Psicología y Tecnologia (LabPsiTec), de la Universidad Jaume I de Castellón y de la Universidad de Valencia, es un grupo de investigación que lleva más de 15 años investigando las posibilidades que las Nuevas Tecnologías de la Información y la Comunicación (TICs) pueden ofrecer a la Psicología Clínica, como herramientas de evaluación y de tratamiento.

En la actualidad, el grupo dispone de un conjunto de aplicaciones que han demostrado eficacia para el tratamiento de las fobias (p, ej. acrofobia, fobia a las arañas, fobia a las cucarachas, miedo a hablar en público, claustrofobia, fobia a volar), el trastorno de pánico con y sin agorafobia, el trastorno de estrés postraumático, el duelo patológico, problemas de la imagen corporal en los trastornos alimentarios, el juego patológico, etc. En concreto, el tratamiento de fobia a animales pequeños mediante el uso del sistema de Realidad Aumentada (RA) es una línea de investigación novedosa y reciente en el campo de las nuevas tecnologías y la Psicología Clínica.

La RA implica incorporar elementos virtuales en el mundo real. Hemos diseñado un programa para reproducir elementos virtuales en el tratamiento de la fobia a los animales pequeños, concretamente para arañas y cucarachas. Durante el tratamiento, en consulta y en un contexto real (una mesa, el suelo, objetos personales del paciente, etc...) se reproducen cucarachas o arañas virtuales. Estos estímulos pueden variar en número, lugar de aparición, tamaño o estado del animal (estático o en movimiento, muerto o vivo) de manera que el paciente pueda exponerse a cada uno de los escenarios generados. La experiencia acumulada en este campo indica que los objetos virtuales (en este caso, animales pequeños) son valorados con gran realismo por los participantes, dado que se encuentran inmersos en el ambiente real.

El estudio que se pretende llevar a cabo pretende someter a prueba, en un estudio controlado, confirmar la eficacia y eficiencia del sistema de RA como herramienta terapéutica para tratar la fobia a animales pequeños.

Introducción

Las fobias específicas son las más prevalentes entre los trastornos de ansiedad. La tasa de prevalencia global es bastante alta (alrededor del 7.2% y 11.3% en población general). La fobia a las cucarachas o a las arañas es un tipo de fobia específica que se clasifica dentro del subtipo de fobia a los animales (APA, 2000). Estas fobias son descritas como un marcado y persistente miedo que es excesivo e irracional, ante la presencia o la anticipación de estos animales. La exposición a estos animales provoca de forma prácticamente invariable una respuesta de ansiedad inmediata, a pesar de que la persona reconoce que este miedo es excesivo e irracional (APA, 2000). En consecuencia, la situación fóbica es evitada o bien la persona la enfrenta experimentando un intenso malestar, de manera que provoca una alta interferencia en la vida de la persona (ámbito familiar, profesional, actividades sociales, etc.).

Actualmente, el tratamiento de elección para las fobias específicas (incluida la fobia a los animales pequeños), es la exposición en vivo. Esto es, un terapeuta, de forma gradual presenta los estímulos temidos al paciente, de manera que se va incrementando la dificultad de las situaciones a las que se expone el paciente, de menor a mayor ansiedad. La mayoría de las personas (60-80%) que padecen fobia nunca buscan tratamiento (Agras, Sylvester & Oliveau, 1969; Boyd et al., 1990; Essau, Conrad, y Petermann, 2000; Magee, Eaton, Wittchen, McGonagle y Kessler, 1996). Además, no todos los pacientes se benefician del tratamiento de exposición en vivo, ya que de entre los pacientes que buscan tratamiento, un alto porcentaje rechazan o abandonan (aproximadamente un 25%) cuando son informados del procedimiento que se va a seguir al aplicar la exposición en vivo (García-Palacios, Botella, Hoffman y Fabregat, 2007; García-Palacios, Hoffman, See, Tsay y Botella, 2001; Marks, 1978, 1992).

Las nuevas tecnologías de la información y la comunicación, tales como la Realidad Virtual (RV) o la Realidad Aumentada (RA) son aplicaciones pioneras que pueden mejorar de forma importante la adherencia al tratamiento y la aceptación del mismo (García-Palacios, Botella, Hoffman y Fabregat, 2007). En estos casos, los pacientes pueden interactuar con ambientes virtuales simulados y relacionados con su miedo, de manera que garantiza la exposición en un contexto seguro y más controlado, con la ayuda del terapeuta.

Hasta el momento, se cuentan con una serie de estudios que ofrecen datos preliminares acerca de la utilidad de las nuevas tecnologías, concretamente de la RA, para el tratamiento de la fobia a los animales pequeños (Botella et al., 2005; Botella, Bretón-López, Quero, Baños & García-Palacios, 2010; Juan et al., 2005). Estos estudios han mostrado que la exposición por medio de RA puede conseguir una importante reducción del temor, la evitación y las creencias asociadas a las situaciones que suponer enfrentarse con estos elementos fóbicos (arañas o una cucarachas). Los participantes que han recibido tratamiento con estos sistemas han mejorado significativamente con el tratamiento y han mantenido sus mejoras en los seguimientos realizados (Botella et al., 2010). Sin embargo, hasta la fecha no contamos con ningún estudio controlado que permita evaluar la eficacia diferencial del uso de las nuevas tecnologías para el tratamiento de la fobia a los animales pequeños frente a la terapia tradicional en vivo (esto es, frente al tratamiento de elección en estos momentos). Por ello, el objetivo de esta investigación es estudiar, por medio de un estudio controlado, la eficacia diferencial de ambas opciones de tratamiento así como las preferencias mostradas por los

participantes, una vez que haya finalizado el tratamiento en cuestión. La hipótesis que se plantea en el estudio es que no existirán diferencias estadísticamente significativas en eficacia entre el tratamiento de exposición en vivo y el tratamiento mediante Realidad Aumentada para la fobia a los animales pequeños; mientras que sí se observarán diferencias estadísticamente significativas respecto a eficiencia entendida como las preferencias de los participantes de un tratamiento o de otro

Metodología

Diseño y muestra del estudio: Se empleará un diseño entre grupos. El tamaño muestra será de, al menos, 50 participantes, 25 asignados al azar a cada una de las condiciones experimentales (exposición en vivo y exposición mediante RA).

Criterios de inclusión: Para ser incluido en el estudio se consideran los siguientes criterios:

1) Tener entre 18 y 60 años de edad.
2) Tener un diagnóstico de fobia a los animales pequeños (cucarachas o arañas) de acuerdo con el DSM-IV.
3) Tener un mínimo de duración de 1 año de la fobia.
4) No tener otro problema psicológico con necesidad inmediata de tratamiento.
5) No padecer enfermedades físicas graves.
6) No padecer en la actualidad dependencia de alcohol o drogas.
7) No estar siendo tratado por un programa de tratamiento similar al que se va a recibir.
8) No estar tomando ansiolíticos durante la duración del estudio (en el caso de que se estén tomando que no se produzcan modificaciones ni el el fármaco ni en la dosis).
9) Aceptar de forma expresa formar parte del estudio.

Protocolo a seguir en el estudio

Previo a la evaluación: Entrevista de admisión (*screening*).

SESIÓN 1: EVALUACIÓN

- Consentimiento informado de participación en el estudio.
- Consentimiento informado de grabación.
- Entrevista estructurada para los Trastornos de Ansiedad (ADIS-IV): Fobia específica.
- Establecer y evaluar conductas objetivo (escala adaptada de Marks y Mathews, 1979).
- Expectativas de cambio de las conductas objetivo.
- Escala de gravedad valorada por el terapeuta.
- Test de evitación conductual.
- Cuestionario de expectativas sobre el tratamiento que van a recibir.

Cuestionarios para cumplimentar al final de la sesión en la sala de terapia:

- Escala de adaptación (Echeburúa y de Corral, 1992).
- Cuestionario de miedo a las cucarachas (adaptado de Zymanski & O'Donohue, 1995).
- Cuestionario de creencias sobre cucarachas (adaptado de SBQ, Arntz, et al. 1993).
- Escala de Asco DPSS-R-12 (Sandin et al., 2008).
- Cuestionario de Depresión. BDI-II (versión 2007).
- Cuestionario de elección exposición RA-Vivo.

SESIÓN 2: TRATAMIENTO

Sesión de tratamiento intensiva de tratamiento en la condición de RA o exposición en vivo. Al finalizar la sesión con RA:

- CPYJR.
- Cuestionario de Presencia (Slater).

Pase del Protocolo Evaluación post-tratamiento para todos los participantes:

- Entrevista estructurada para los Trastornos de Ansiedad (ADIS-IV): Fobia específica.
- Evaluar conductas objetivo (escala adaptada de Marks y Mathews, 1979).
- Escala de gravedad valorada por el terapeuta.
- Escala de mejoría de la fobia (valorado por terapeuta).
- Test de evitación conductual.
- Cuestionario de satisfacción acerca del tratamiento que han recibido.
- Cuestionario de preferencias de tratamiento en vivo versus RA.

Cuestionarios para cumplimentar al final de la sesión en la sala de terapia:

- Escala de adaptación (Echeburúa y de Corral, 1992).
- Cuestionario de miedo a las cucarachas (adaptado de Zymanski & O'Donohue, 1995).
- Cuestionario de creencias sobre cucarachas (adaptado de SBQ, Arntz, et al1993).
- Escala de Asco DPSS-R-12 (Sandin et al., 2008).
- BDI-II (versión 2007).

SEGUIMIENTOS (1, 3, 6 y 12 meses):

- Entrevista estructurada para los Trastornos de Ansiedad (ADIS-IV): Fobia específica.
- Evaluar conductas objetivo (escala adaptada de Marks y Mathews, 1979).
- Escala de gravedad valorada por el terapeuta.
- Escala de mejoría de la fobia (valorado por terapeuta).
- Test de evitación conductual.
- Cuestionario de satisfacción sobre el tratamiento que han recibido.
- Escala de adaptación (Echeburúa y de Corral, 1992).

- Cuestionario de miedo a las cucarachas (adaptado de Zymanski & O'Donohue, 1995).
- Cuestionario de creencias sobre cucarachas (adaptado de SBQ, Arntz, et al. 1993).
- Escala de Asco DPSS-R-12 (Sandin et al., 2008).
- BDI-II (versión 2007).
- Cuestionario de elección exposición RV-Vivo.

Análisis estadístico de los datos

Se utilizará el programa estadístico SPSS v.17 y se utilizarán los procedimienots estadísticos y las estrategias que resulten pertienentes para el análisis de los datos (MANOVAS, ANOVAS, consideración del tamaño de los efectos, análisis del cambio clínicamente significativo etc.) de manera que se pueda determinar si se han cumplido o no las hipóstesis que se plantean en el estudio.

Implicaciones del estudio

El presente estudio supone analizar en profundidad y de una forma rigurosa la eficacia de las nuevas tecnologías, en concreto la RA, frente al tratamiento tradicional, para abordar el tratamiento de una fobia específica. La comparación entre ambos tratamientos, así como las preferencias manifestadas por los participantes supondrá un avance importante en cuanto a los beneficios que puede otorgar una tecnología de estas características para tratar problemas que generan una importante interferencia en la vida de las personas.

Los resultados pretenden aportar información acerca de la utilidad clínica de los actuales desarrollos tecnológicos para la mejora de la eficacia y eficiencia de los tratamientos psicológicos basados en la evidencia. Por último, mencionar que hasta el momento y de acuerdo con la revisión teórica, es el primer estudio controlado que analiza la eficacia y la eficiencia diferencial de ambos tratamientos, por lo que se puede convertir en una aportación muy relevante a la literatura existente en relación a esta temática.

Bibliografía

Agras, S., Sylvester, D., y Oliveau, D. (1969). The epidemiology of common fears and phobia. *Comprehensive Psychiatry, 10,* 151-156.

American Psychiatric Association, APA (2000). *Diagnostic and statistical manual for mental disorders (4ª edition, revised text) (DSM-IV-TR).* Washington, DC: Autor.

Botella, C., Bretón-López, J., Quero, S., Baños, R. y García-Palacios, A. (2010). Treating Cockroach Phobia With Augmented Reality. *Behavior Therapy, 41,* 401-413.

Botella, C., Juan, M.C., Baños, R., Alcañiz, M., Guillén, V., y Rey, B. (2005). Mixing realities? an application of augmented reality for the treatment of cockroach phobia. *CyberPsychology & Behavior, 8,* 162-171.

Boyd, J.H., Rae, D.S., Thompson, J.W., Burns, B.J., Bourdon, K., Locke, B.Z., et al. (1990). Phobia: prevalence and risk factors. *Social Psychiatrica and Psychiatric Epidemiology, 25,* 314-323.

Essau, C.A., Conradt, J., y Petermann, F. (2000). Frequency, comorbidity and psychosocial impairment of specific phobia in adolescents. *Journal of Clinical Child Psychology, 29,* 221-231.

García-Palacios, A., Botella, C., Hoffman, H., y Fabregat, S. (2007). Comparing acceptance and refusal rates of virtual reality exposure vs. in vivo exposure by patients with specific phobias. *CyberPsychology & Behavior, 10,* 722-724.

García-Palacios, A., Hoffman, H.G., See, S.K., Tsay, A., y Botella, C. (2001). Redefining therapeutic success with virtual reality exposure therapy. *CyberPsychology & Behavior, 4,* 341-348.

Juan, M. C., Alcañiz, M., Monserrat, C., Botella, C., Baños, R., y Guerrero, B. (2005). Using augmented reality to treat phobias. *IEEE Computer Graphics and Applications, 25,* 31-37.

Magee, W., Eaton, W.W., Wittchen, H.U., McGonagle, K.A., y Kessler, R.C. (1996). Agoraphobia, simple phobia, and social phobia in the national comorbidity survey. *Archives of General Psychiatry, 53,* 159-168.

Marks, I.M. (1978). Behavioral psychotherapy of adult neurosis. In S.L. Gardfield & A.E. Bergin (eds.), *Handbook of psychotherapy and behaviour change*, (pp. 493-598). New York: Wiley.

Marks, I.M. (1992). Tratamiento de exposición en la agorafobia y el pánico. In E. Echeburúa (ed.), *Avances en el tratamiento psicológico de los trastornos de ansiedad* (pp. 35-55). Madrid: Pirámide.

HOJA CONSENTIMIENTO INFORMADO

El/La abajo firmante, D. Dª
…………………………………………………………………..
ha sido informado acerca de los siguientes puntos:

- La participación en este proyecto es totalmente voluntaria y puedo abandonarlo en el momento que desee sin ningún tipo de consecuencia.
- El objetivo de este proyecto es intentar mejorar la comprensión del diagnóstico y tratamiento del miedo a los animales pequeños (arañas y cucarachas), así como mejorar los procedimientos diagnósticos y terapéuticos para estos problemas.
- La participación en este proyecto no supone ningún riesgo para la salud física y mental.
- Si sufro de miedo a alguno de los animales mencionados podré participar en estudio centrado en la aplicación de un tratamiento psicológico para ayudarme a superar este problema.
- Se salvaguardará siempre mi derecho a la intimidad y la confidencialidad de la información que proporcione.

Una vez que he sido informado/a de todo lo anterior y se me ha respondido a todas las preguntas o dudas que haya podido tener, doy mi consentimiento voluntario para que los datos que se deriven de mi participación en este proyecto sean publicados en ámbitos de divulgación científica.

Lo que declaro con fecha……………………………….

Firmado……………………………….

HOJA CONSENTIMIENTO GRABACIONES EN VIDEO / AUDIO

Nombre y Apellidos: ____

Uno de los Recursos de Servicio de Asistencia psicológica de la Universidad Jaume I, es la grabación de las sesiones en vídeo y/o audio. Las grabaciones proporcionan una información muy valiosa que se utiliza:

- Como herramienta terapéutica (p. ej. en algún momento del tratamiento el terapeuta puede pedir al paciente como tarea para casa visionar una o varias sesiones)

- Para analizar y trabajar en el proceso terapéutico de un caso concreto por parte de los distintos terapeutas que componen el Servicio de Asistencia Psicológica

- Para la formación en cursos de postgrado (para licenciados en psicología/medicina) y en congresos especializados. En este caso, cuando la grabación es en vídeo se procede a la distorsión de las imágenes y se elimina todo aquello que pudiera dar lugar a la identificación de la persona.

MANIFIESTO:

Que he sido informado adecuadamente de la posible utilización de las grabaciones de las sesiones que se realizarán durante el tratamiento psicológico que recibiré en el Servicio de Asistencia Psicológica de la Universidad Jaume I

Que, salvaguardando siempre mi derecho a la intimidad y al anonimato, estoy de acuerdo y acepto libre y voluntariamente la grabación en video/ audio de las sesiones de terapia.

Que, en función de esta opción libre, puedo cambiar mi opinión y pedir que no se lleve a cabo la grabación de las sesiones en el momento que lo desee.

El interesado/a,

La Directora del Servicio de Asistencia Psicológica de la Universidad Jaume I

Castellón, _____ de _______________ del 201_____

Servicio de Asistencia Psicológica: Protección de datos y aspectos éticos

El Servicio de Asistencia Psicológica de la Universitat Jaume I sigue los principios de la declaración de Helsinki y de la Convención Europea sobre los Derechos Humanos y la Biomedicina. Además, las investigaciones que se llevan a cabo siguen el Código Deontológico establecido por el Colegio Oficial de Psicólogos y se ajustan a la Ley de protección de datos, Ley Orgánica 15/1999.

En el SAP se siguen las siguientes medidas para la protección de los datos:

- Existe una sala de archivo exclusivo para custodiar la historia clínica de los pacientes. Esta sala de archivo posee una cerradura electrónica que registra los accesos (persona, día y hora), además los expedientes se encuentran en archivadores de metal con llave, que es custodiada por la administrativa. En el archivo se custodian todos los datos relativos a pacientes que han pasado por el SAP desde su puesta en funcionamiento en la UJI, en diciembre de 1993.

- Únicamente tres miembros del SAP disponen del acceso a esta sala de archivo: coordinadora clínica, administrativa y responsable de equipos e infraestructura. La forma de acceso de los terapeutas a los expedientes clínicos, es solicitar a la administrativa el expediente. Tras la consulta la administrativa repone el expediente en la sala de archivo.

- A los pacientes se les informa verbalmente y por escrito de que sus datos personales serán tratados de forma confidencial. La información escrita incluye la petición de permiso para que los datos recogidos durante la evaluación y el tratamiento puedan utilizarse con fines de investigación, salvaguardando siempre el derecho a la intimidad y el anonimato.

- En todos los estudios llevados a cabo en el SAP se incluye información verbal y por escrito (hojas de consentimiento) para los participantes, también se hace explícito que las investigaciones se adecuan a los estándares éticos nacionales e internacionales (por ejemplo, código deontológico del COP, de la APA, Declaración de Helsinki).

- Todo el personal del servicio está sujeto al código deontológico que incluye la salvaguarda del derecho a la intimidad y al anonimato de los pacientes.

- En relación al soporte informatizado: la creación de bases de datos con información de los pacientes respecto a la evaluación y los resultados del tratamiento no contiene en ningún caso datos personales identificativos del paciente. En su lugar, a cada participante se le asigna un código numérico. La relación entre el código numérico y el participante sólo se conserva en papel y es tratado como un expediente clínico, custodiándose en la sala de archivo.

- En cuanto al manejo de los datos, se utiliza SPSS para el tratamiento estadístico de los datos de los pacientes, utilizando códigos numéricos para la asociación con los pacientes. A partir de ese momento los terapeutas deben trabajar sólo con el código numérico. En ningún caso aparecen los nombres ni información que permita la identificación de los pacientes.

- En relación al material audiovisual generado (vídeo y audio) este es almacenado en un armario con llave, en una sala de control audiovisual, que cuenta con cerradura electrónica y que registra los accesos a la misma. La llave de dicho armario está custodiada por la persona responsable de equipos e infraestructura.
