# Supplementary material for: In Vivo versus Augmented Reality Exposure in the Treatment of Small Animal Phobia: A Randomized Controlled Trial
Source: PLoS One. 2016 Feb 17;11(2):e0148237. doi: 10.1371/journal.pone.0148237 (PMC4757089; doi:10.1371/journal.pone.0148237)
Supplement: S3 File — (PDF) [file pone.0148237.s004.pdf]

Beatriz Tomás Mallén, secretaria de la Comisión Deontológica de la Universitat Jaume I de Castelló de la Plana,

CERTIFICA: Que la Comisión Deontológica de la Universitat Jaume I ha emitido informe favorable sobre el procedimiento de investigación titulado: "La eficacia del uso de la terapia tradicional (exposición en vivo) versus la exposición por medio de realidad aumentada para el tratamiento de la fobia a los animales pequeños", cuya investigadora principal es Cristina Botella Arbona, por considerar que cumple las normas deontológicas exigidas.

Castellón de la Plana, 19 de enero de 2011
