# Supplementary material for: In Vivo versus Augmented Reality Exposure in the Treatment of Small Animal Phobia: A Randomized Controlled Trial
Source: PLoS One. 2016 Feb 17;11(2):e0148237. doi: 10.1371/journal.pone.0148237 (PMC4757089; doi:10.1371/journal.pone.0148237)
Supplement: S1 Table — (DOCX) [file pone.0148237.s005.docx]

**S1 Table. Means, standard deviations, within-group and between-group effect sizes for the ITT analysis of all outcome measures at 3-month follow-up.**

| **Measure and group** | | **3-month FU** | **Within-group**  **effect size pre-3-month FU** | **Between group effect size at 3-month FU** |
| --- | --- | --- | --- | --- |
|  |  | **Mean (SD)** | ***d* (95% CI)** | ***d* (95% CI)** |
| **BAT** | Fear-BAT |  |  |  |
|  | IVE | 2.84 (2.67) | 2.26 (1.64 to 2.87) | -0.38 (-1.09 to 0.32) |
|  | ARE | 3.94 (3.12) | 1.78 (1.14 to 2.43) |  |
|  | Avoidance-BAT | |  |  |
|  | IVE | 2.23 (2.93) | 2.63 (2.00 to 3.26) | -0.28 (-1.14 to 0.57) |
|  | ARE | 3.22 (4.04) | 1.67 (0.90 to 2.45) |  |
|  | Belief-BAT | |  |  |
|  | IVE | 1.90 (2.64) | 2.38 (1.70 to 3.06) | -0.28 (-0.95 to 0.39) |
|  | ARE | 2.66 (2.87) | 2.36 (1.74 to 2.98) |  |
|  | Performance-BAT | |  |  |
|  | IVE | 10.26 (2.13) | -2.89 (-3.42 to -2.36) | 0.39 (-0.28 to 1.05) |
|  | ARE | 9.22 (3.21) | -1.94 (- 2.59 to -1.29) |  |
|  | Maximun anxiety-BAT | |  |  |
|  | IVE | 3.10 (2.24) | 1.95 (1.44 to 2.46) | -0.21 (-0.79 to 0.38) |
|  | ARE | 3.59 (2.55) | 1.61 (1.08 to 2.14) |  |
|  | Severity-BAT (Clinician) | |  |  |
|  | IVE | 2.00 (1.46) | 2.85 (2.52 to 3.18) | -0.39 (-0.88 to 0.10) |
|  | ARE | 2.78 (2.46) | 1.54 (1.08 to 2.01) |  |
| **FSQ** | IVE | 34.68 (25.03) | 3.07 (-1.90 to 8.03) | -0.28 (-7.30 to 6.74) |
|  | ARE | 42.56 (32.20) | 2.01 (-4.28 to 8.30) |  |
| **SBQ** | SBQ-1 |  |  |  |
|  | IVE | 16.11 (13.04) | 2.76 (-0.51 to 6.04) | -0.22 (-4.28 to 3.84) |
|  | ARE | 19.70 (19.62) | 1.60 (-2.94 to 6.14) |  |
|  | SBQ-2 |  |  |  |
|  | IVE | 7.21 (7.48) | 2.34 (-1.13 to 5.82) | -0.43 (-4.27 to 3.40) |
|  | ARE | 13.90 (20.88) | 1.22 (-3.82 to 6.26) |  |
| **MTB** | Fear-MTB |  |  |  |
|  | IVE | 2.65 (2.63) | 2.86 (2.34 to 3.38) | -0.25 (-0.90 to 0.40) |
|  | ARE | 3.31 (2.72) | 2.60 (2.09 to 3.12) |  |
|  | Avoidance-MTB | |  |  |
|  | IVE | 1.97 (3.24) | 2.80 (2.17 to 3.43) | -0.26 (-1.04 to 0.52) |
|  | ARE | 2.78 (3.19) | 2.41 (1.80 to 3.02) |  |
|  | Belief -MTB | |  |  |
|  | IVE | 2.55 (2.61) | 2.57 (1.99 to 3.16) | -0.17 (-0.82 to 0.49) |
|  | ARE | 3.00 (2.74) | 2.73 (2.22 to 3.25) |  |
| **CSS** | IVE | 2.07 (1.65) | 2.59 (2.25 to 2.93) | -0.36 (-0.78 to 0.06)) |
|  | ARE | 2.69 (1.82) | 2.27 (1.92 to 2.62) |  |

^a^MTB = Main Target Behaviour

^b^CSS = Clinician Severity Scale.
