# Supplementary material for: In Vivo versus Augmented Reality Exposure in the Treatment of Small Animal Phobia: A Randomized Controlled Trial
Source: PLoS One. 2016 Feb 17;11(2):e0148237. doi: 10.1371/journal.pone.0148237 (PMC4757089; doi:10.1371/journal.pone.0148237)
Supplement: S2 Table — (DOCX) [file pone.0148237.s006.docx]

**S2 Table. Clinically Meaningful Improvement on the FSQ and SBQ Scores at Post-treatment, 3- and 6-month follow-up.**

|  | **Post-treatment** | |  | **3-month FU^a^** | |  | **6-month FU^a^** | |
| --- | --- | --- | --- | --- | --- | --- | --- | --- |
| **Measures** | **IVE** | **ARE** |  | **IVE** | **ARE** |  | **IVE** | **ARE** |
| **FSQ** |  |  |  |  |  |  |  |  |
| Recovered | 27(87.1%) | 29(90.6%) |  | 27(87.1%) | 24(75.0%) |  | 24(77.4%) | 25(78.1%) |
| Improved | 3 (9.7%) | 2(6.3%) |  | 3 (9.7%) | 5(15.6%) |  | 5 (16.1%) | 4(12.5%) |
| No change | 0 (0%) | 1(3.1%) |  | 1 (3.2%) | 3(9.4%) |  | 1 (3.2%) | 3(9.4%) |
| Impaired | 1 (3.2%) | 0(0%) |  | 0(0%) | 0(0%) |  | 1 (3.2%) | 0(0%) |
| **SBQ-1** |  |  |  |  |  |  |  |  |
| Recovered | 25(80.6%) | 24(75.0%) |  | 25(80.6%) | 24(75.0%) |  | 25(80.6%) | 26(81.3%) |
| Improved | 6 (19.4%) | 5 (15.6%) |  | 6 (19.4%) | 5 (15.6%) |  | 6 (19.4%) | 3 (9.4%) |
| No change | 0 (0%) | 3 (9.4%) |  | 0 (0%) | 3 (9.4%) |  | 0 (0%) | 3 (9.4%) |
| Impaired | 0 (0%) | 0(0%) |  | 0 (0%) | 0 (0%) |  | 0 (0%) | 0 (0%) |
| **SBQ-2** |  |  |  |  |  |  |  |  |
| Recovered | 19(61.3%) | 18(56.3%) |  | 25(80.6%) | 18(56.3%) |  | 22(71.0%) | 20(62.5%) |
| Improved | 10(32.3%) | 12(37.5%) |  | 6 (19.4%) | 9 (28.1%) |  | 6 (19.4%) | 8 (25.0%) |
| No change | 1 (3.2%) | 2 (6.3%) |  | 0 (0%) | 4 (12.5%) |  | 2 (6.5%) | 3 (9.4%) |
| Impaired | 1 (1.6%) | 0 (0%) |  | 0 (0%) | 1 (3.1%) |  | 1 (1.6%) | 1 (1.6%) |

^a^FU: Follow-up
