# Supplementary material for: In Vivo versus Augmented Reality Exposure in the Treatment of Small Animal Phobia: A Randomized Controlled Trial
Source: PLoS One. 2016 Feb 17;11(2):e0148237. doi: 10.1371/journal.pone.0148237 (PMC4757089; doi:10.1371/journal.pone.0148237)
Supplement: S3 Table — (DOCX) [file pone.0148237.s007.docx]

**S3 Table. Expectations and satisfaction with the exposure component.**

|  |  | **Expectations** | | | **Satisfaction** | | |
| --- | --- | --- | --- | --- | --- | --- | --- |
| **Measures** | **Group** | **Mean (SD)** | **t** | ***p*** | **Mean (SD)** | ***t*** | ***p*** |
| Logical | IVE | 9.03 (1.68) | 0.566 | 0.573 | 9.45 (0.81) | 0.677 | 0.501 |
|  | ARE | 8.81 (1.38) |  |  | 9.31 (0.82) |  |  |
| Satisfaction | IVE | 7.21 (3.67) | -1.560 | 0.124 | 9.23 (1.09) | 1.204 | 0.233 |
|  | ARE | 8.34 (1.70) |  |  | 8.78 (1.75) |  |  |
| Recommend to  others | IVE | 8.32 (2.80) | -0.981 | 0.330 | 9.52 (1.12) | 1.097 | 0.277 |
|  | ARE | 8.88 (1.43) |  |  | 9.09 (1.84) |  |  |
| Utility for other  problems | IVE | 8.29 (1.86) | -0.055 | 0.956 | 9.19 (1.11) | 1.486 | 0.143 |
|  | ARE | 8.31 (1.89) |  |  | 8.59 (1.96) |  |  |
| Utility for patient’s problem | IVE | 8.18 (2.55) | 0.319 | 0.751 | 9.16 (1.10) | 2.326 | **0.023^a^** |
|  | ARE | 8.00 (1.76) |  |  | 8.22 (1.98) |  |  |
| Aversiveness | IVE | 8.14 (2.52) | 2.263 | **0.027^a^** | 6.45 (2.49) | 4.369 | **0.000^b^** |
|  | ARE | 6.69 (2.45) |  |  | 3.47 (2.91) |  |  |

^a^ <0.05

^b^< 0.001
